# Unraveling the Reaction Mechanism of Russell’s Viper Venom Factor X Activator: A Paradigm for the Reactivity of Zinc Metalloproteinases?

**DOI:** 10.1021/acs.jcim.2c01156

**Published:** 2023-04-24

**Authors:** Juliana Castro-Amorim, Ana Oliveira, Ashis K. Mukherjee, Maria J. Ramos, Pedro A. Fernandes

**Affiliations:** † LAQV, REQUIMTE, Departamento de Química e Bioquímica, Faculdade de Ciências, Universidade do Porto, Rua do Campo Alegre, s/n, Porto 4169-007, Portugal; ‡ Institute of Advanced Study in Science and Technology, Vigyan Path Garchuk, Paschim Boragaon, Guwahati 781035, Assam, India

## Abstract

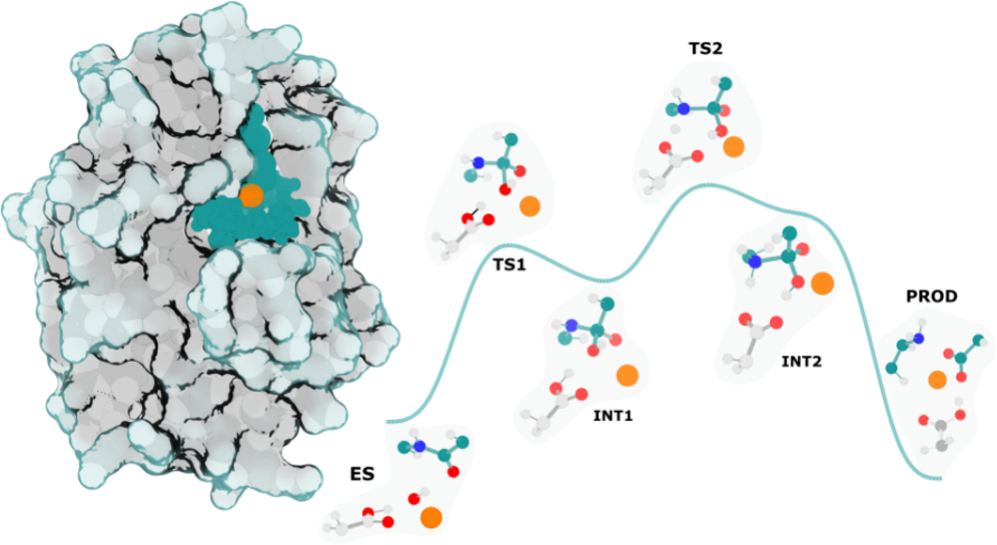

Snake venom metalloproteinases (SVMPs) are important drug targets against snakebite envenoming, the neglected tropical disease with the highest mortality worldwide. Here, we focus on Russell’s viper (*Daboia russelii*), one of the “big four” snakes of the Indian subcontinent that, together, are responsible for ca. 50,000 fatalities annually. The “Russell’s viper venom factor X activator” (RVV-X), a highly toxic metalloproteinase, activates the blood coagulation factor X (FX), leading to the prey’s abnormal blood clotting and death. Given its tremendous public health impact, the WHO recognized an urgent need to develop efficient, heat-stable, and affordable-for-all small-molecule inhibitors, for which a deep understanding of the mechanisms of action of snake’s principal toxins is fundamental. In this study, we determine the catalytic mechanism of RVV-X by using a density functional theory/molecular mechanics (DFT:MM) methodology to calculate its free energy profile. The results showed that the catalytic process takes place via two steps. The first step involves a nucleophilic attack by an in situ generated hydroxide ion on the substrate carbonyl, yielding an activation barrier of 17.7 kcal·mol^–1^, while the second step corresponds to protonation of the peptide nitrogen and peptide bond cleavage with an energy barrier of 23.1 kcal·mol^–1^. Our study shows a unique role played by Zn^2+^ in catalysis by lowering the p*K*_a_ of the Zn^2+^-bound water molecule, enough to permit the swift formation of the hydroxide nucleophile through barrierless deprotonation by the formally much less basic Glu140. Without the Zn^2+^ cofactor, this step would be rate-limiting.

## Introduction

### Snakebite Epidemiology

Snakebite envenoming represents a public health hazard affecting tropical countries’ rural populations.^1^ Tropical and subtropical regions of the world, like Asia, sub-Saharan Africa, Latin America, and parts of Oceania, suffer the most significant impact.^2−4^ Although the actual number of people bitten by snakes is not known accurately,^5^ estimates suggest that, per year, approximately 5.4 million snakebites occur, with up to 2.7 million cases (one-half) leading to snakebite envenoming, which causes 81,000–138,000 deaths and around 400,000 amputations and permanent sequelae.^1,3,6^ Despite being globally distributed, snakebite envenoming in India is the highest compared to any other country,^4^ accounting for nearly half of global snakebite deaths.^5,7^

In India, most of the snakebites are inflicted by the “big four” snakes, which include the saw-scaled viper (*Echis carinatus*), the spectacled cobra (*Naja naja*), the common krait (*Bungarus caeruleus*), and the Russell’s viper (*Daboia russelii*).^4,5,8,9^ The latter, which will be further discussed, is one of the major venomous snakes responsible for the high rates of morbidities and mortality in the Indian subcontinent and Southeast Asia,^7,10−13^ representing a severe medical threat to the local population. Unfortunately, access to health services is limited in the remote rural areas where most snakebites occur.^6,14^ The only reasonably efficient therapy is antibody-based antivenom,^4,6,14,15^ which is very expensive and needs a cold chain of transportation and storage, and inpatient administration as it is frequently associated with anaphylactic reactions.^14,15^ Therefore, the therapy is of meager availability to those in desperate need.^4,14−16^

Following several requests, in 2017, the World Health Organization (WHO) recognized snakebite envenoming as a neglected tropical disease (NTD),^3,4,14,17^ presently the one leading to the most significant number of annual deaths. Therefore, in May 2019, the WHO launched a work plan outlining the goal of halving mortality and disabilities caused by envenoming by 2030.^3,14,18^ Among other measures, this action plan promotes the research and development of inexpensive, heat-stable, affordable-for-all small-molecule inhibitors that can be stored in local villages and administered outside a hospital settlement.^3,18^ Some chelators have offered great potential for snakebite treatment by inhibiting snake venom metalloproteinase (SVMP) toxins, including the peptidomimetic hydroxamate inhibitor (WR2), ilomastat, marimastat, and batimastat. Although initially designed for inhibiting human extracellular matrix metalloproteinases (MMPs), these were used for repurposing based on the high degree of sequence identity shared by MMPs and SVMPs. However, all failed in the clinical trials due to toxicity problems brought on by off-target effects.^19,20^

### Russell’s Viper and Its Venom

This paper focuses on one of the leading actors of the problem, Russell’s viper (RV), a large and beautiful snake that is responsible for most snakebites that occur in the vicinity of the agricultural lands and villages^4,9,12,21,22^ where its primary dietary preference, rats and mice, grow.^5,8^ The RV is a severe issue for rice cultivators due to its abundance in rice paddies and for other farmers that work in its habitat and therefore are its primary victims,^4,5,8^ as the snake usually reacts to human presence by concealing itself (*Daboia* means “the lurker” in Hindi) but bites if the farmer incidentally steps on it.^4^

**Figure 1:**
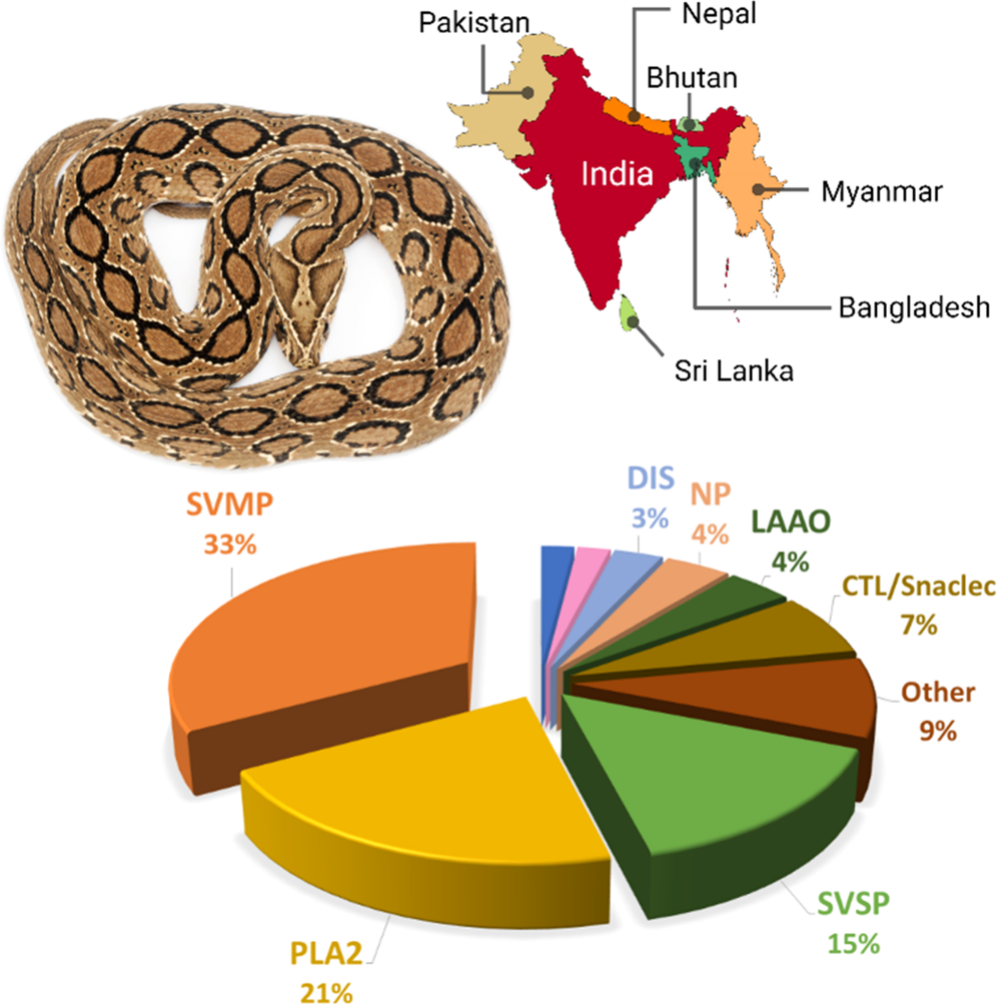
Average proteomic profile of Russell’s Viper venom. The principal protein families are the l-amino acid oxidase (LAAO), natriuretic peptides (NP), serine protease (SVSP), phospholipase A_2_ (PLA_2_), snake C-type lectins (CTL/Snaclec), metalloproteinases (SVMP), and disintegrin (DIS).^1^

Snake venoms have an essentially proteinaceous nature.^5^ They are composed of up to 200 different proteins, and Russell’s viper venom (RVV) is no exception. The enzymatic toxins phospholipases A_2_, metalloproteinases, and serine proteases constitute most of Russell’s viper venom (Figure 1; see ref (1) for a review on snake venom composition). These enzymes are responsible for several local and systemic clinical manifestations, including edema, tissue necrosis, hemorrhage, acute renal failure, and hypotension.^8,11,14,23^ Besides those, some events of neurotoxicity and myotoxicity post RV envenomation have been documented in Sri Lankan and South Indian populations^11^ and Myanmar, respectively.^8,10,24^ Envenomation further reduces male sex hormones; the complete loss of libido and secondary sexual characteristics (for example, loss of facial hair) and even reduction of the size of the sexual organs are not uncommon. Recovery is rarely complete.^25^

### Structure and Function of Metalloproteinases

SVMPs are zinc-dependent metalloproteinases with a modular structure that might include in the most complex isoforms (such as in RVV-X) a metalloproteinase, a disintegrin-like, a cysteine-rich, and a C-type lectin-like domain.^26−30^ The metalloproteinase domains are characterized by a strictly conserved catalytic consensus sequence HEXXHXXGXXH, whose three histidine residues coordinate to the zinc ion through its Nε2 atoms.^19,27,30,31^ A water molecule completes the tetrahedral coordination of the cofactor and is hydrogen-bonded to Glu140.^32−34^

### RVV-X

**Figure 2:**
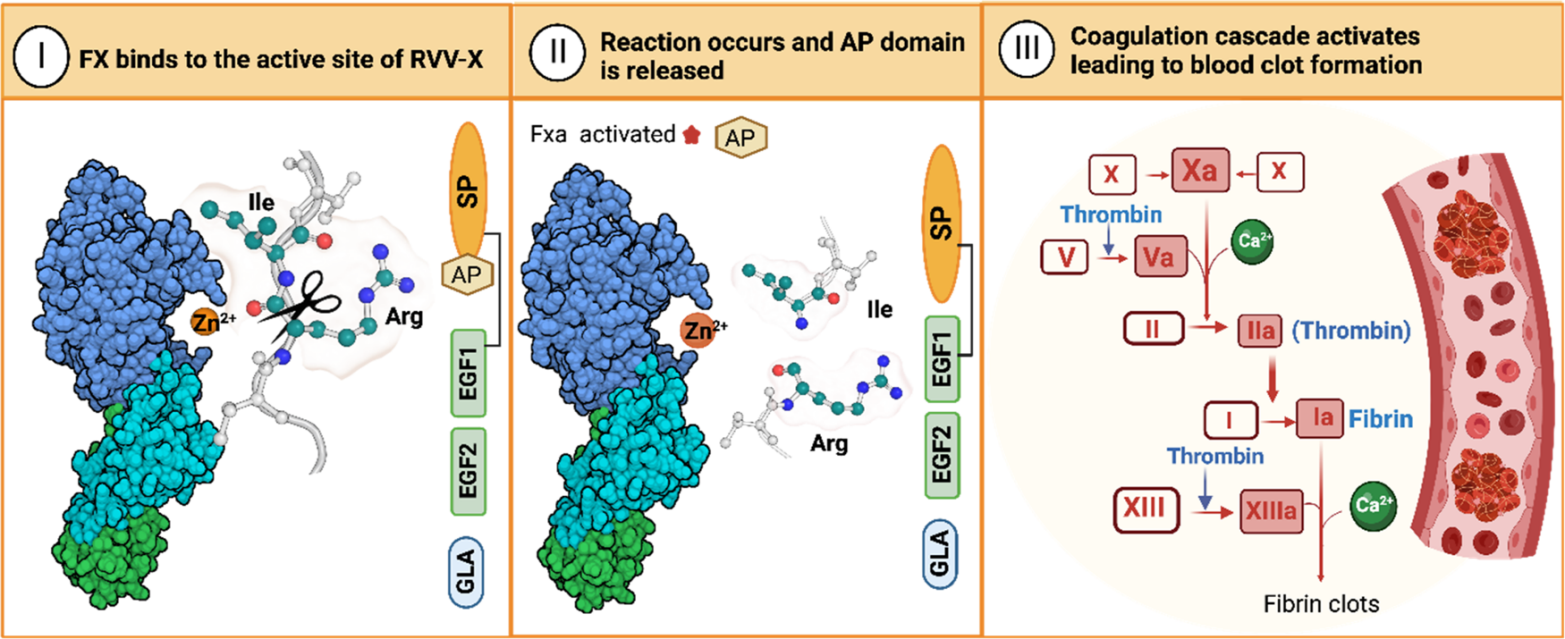
Schematic model of the FX conversion to its active form, FXa, by the RVV-X. (I) The FX activation peptide binds to the active site of the RVV-X; (II) the Arg152–Ile153 scissile bond is cleaved by the RVV-X, resulting in the release of the 52-residue activation peptide; (III) consequently, the common coagulation pathway is activated, leading to the formation of blood clots. The stereo structure of the RVV-X is colored by chain (HC: dark blue, LC1: green, LC2: cyan) and shown in VDW representation. The orange circle represents the zinc ion. Created with BioRender.com.

In some geographic regions, RVV exhibits potent anticoagulant effects, whereas, in others, it exhibits potent pro-coagulant activity.^8,10,12,35^ The latter is mainly due to a specific toxin of its venom, RVV-X,^8,10,36^ which is the most potent venom coagulation activator known that causes fatal envenomation.^36^ It is a non-hemorrhagic class III (subclass d) snake venom metalloproteinase^10^ (Supporting Information, Figure S1) that interferes in the victim’s hemostasis by triggering uncontrolled coagulation through activation of the coagulation factor X (FX).^8,36^ The activation is triggered by the RVV-X catalytic cleavage of a specific peptide bond between Arg152 and Ile153 (human numbering) of FX, resulting in the release of a 52-residue peptide named as “activation peptide” (Figure 2).^8,21,34,37^ The cleavage leads to the conversion of the zymogen FX into its active form FXα and, consequently, to the coagulation cascade’s activation.^21,34,36−38^ Thus, just like for other vipers, SVMPs play a determinant role in the pathogenesis of RV bites.^39^

The structure of RVV-X has already been solved by X-ray crystallography. Takeda and co-workers determined its complete structure in 2007 with a resolution of 2.91 Å.^40^ RVV-X is a 93 kDa heterotrimeric glycoprotein composed of three disulfide-linked glycosylated polypeptide chains, one heavy chain (HC), and two light chains (LC1 and LC2). The backbone structure of the HC (α-chain, 57.6 kDa) follows the characteristics of class III SVMPs with its three metalloproteinase, disintegrin-like, and Cys-rich domains. These domains are organized in a C-shape configuration where the metalloproteinase domain interacts with the Cys-rich domain. The latter interacts with the LC1 via a disulfide bond.^8,27,36,40^ The LC subunit (β- and γ-chains, 19.4 and 16.4 kDa) forms a domain-swapped dimer. The light chains share significant sequence identity with snake venom C-type lectin-like proteins. The dimeric interface formed by the two LC is a concave structure that may function as an exosite that confers both affinity and binding specificity for the Gla domain of FX in the presence of Ca^2+^ ions.^36,39,41^

Due to its high specificity, RVV-X is a remarkable biotechnological tool widely used in coagulation research and medical diagnostics.^5,31,42^ For example, the Stypven time is determined by a clotting assay that measures the FX to FXa conversion by RVV-X and subsequent prothrombin activation that initiates clot formation.^43−45^ In particular, a long Stypven time is a diagnostic of FX deficiency, also called Stuart–Prower factor deficiency.^44^

### Catalytic Mechanism of SVMP

**Scheme 1:**
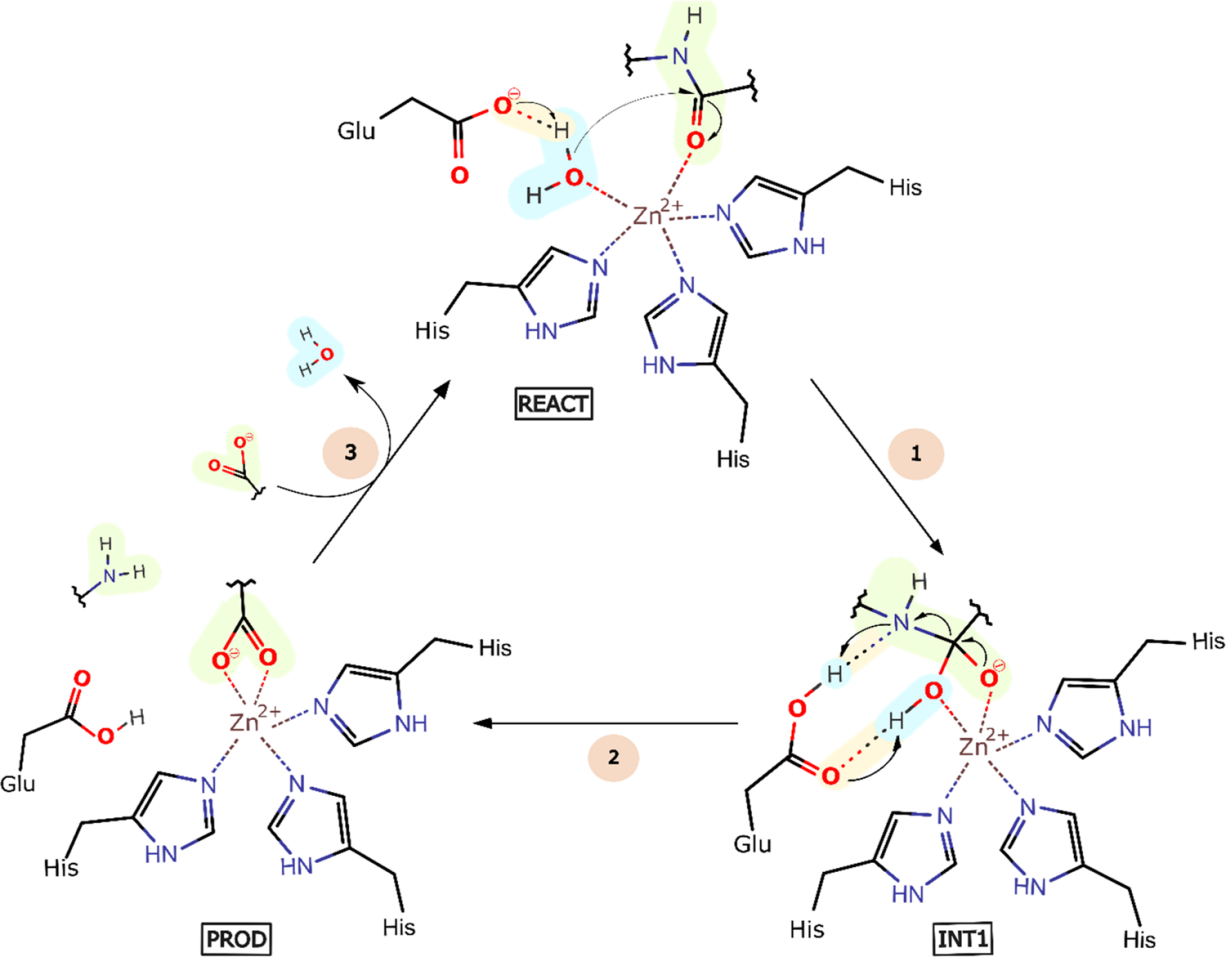
“Water-Promoted Pathway,” A Plausible Catalytic Mechanism for the SVMPs (1) The conserved Glu acts as a base and deprotonates the Zn^2+^-bound water molecule. The generated hydroxide ion attacks the Zn^2+^-bound peptide carbonyl, generating a Zn^2+^-stabilized gem-diolate; the oxygen from the Zn^2+^-bound substrate carbonyl becomes negatively charged; collapse of TS1 into INT1 and hydrogen-bond network rearrangement of the conserved Glu; (2) the neutral Glu protonates the amine nitrogen of the scissile substrate bond, breaking the peptide bond; the proton from the hydroxyl group of the peptide is also shuttled to the amine product by the Glu residue or, alternatively, the proton comes from the bulk solvent after the product release; releasing the first fragment of the products, an amine-terminated peptide; (3) the gem-diolate unbinds the Zn^2+^ ion, through replacement by a water molecule, generating the second fragment of the product, a carboxylic-acid-terminated peptide and regenerating the active site. Alternatively, the water deprotonation can precede the hydrolysis, with the active site ground state consisting of a hydroxide-bound Zn^2+^ ion and a neutral Glu, which is plausible given the lowering of the water p*K*_a_ that binding the metal provides. In this scenario, the peptide bond’s attack and the protonation of the amine nitrogen can occur in a concerted fashion or sequentially.

The reaction mechanism of SVMPs has not been elucidated so far.^26,46^ Typically, snake venom metalloproteinase X-ray structures show the Zn^2+^ cofactor coordinated by the three His residues of the catalytic motif and sometimes by a water molecule^33,47−50^ or a doubly coordinated peptidomimetic inhibitor^40,51−54^ replacing both the water molecule and the substrate. Computational studies of the reaction mechanism of the homologous human matrix metalloproteinases (MMPs) carried out by Pelmenschikov and Siegbahn,^55^ Díaz and Suárez,^56^ and Vasilevskaya et al.^57^ shed some light on the possible reaction mechanism of SVMP. The former group studied the hydrolysis of *N*-methylacetamide catalyzed by the matrix metalloproteinase-3 using a two-layered hybrid of our own n-layered Integrated molecular orbital and molecular mechanics (ONIOM) method, while the last two groups studied the hydrolysis of peptides by the matrix metalloproteinase-2. These studies point to a mechanism for the human MMPs named the “water-promoted pathway”^58−60^ shown in Scheme 1, providing a starting point for the understanding of the reaction mechanism of the SVMPs.

Mutations in the highly conserved catalytic residues affect one or more stages of the catalytic process.^32,33^ For example, in 1994, Crabbe et al.^61^ concluded through mutation experiments that the glutamate residue plays an essential role in the MMP-2 reaction, as its substitution by an alanine led to a marked decrease in the enzymatic activity.

Nevertheless, metalloproteinases are versatile in terms of their catalytic mechanisms. Besides the water-promoted pathway, there is evidence of an alternative mechanism in other related zinc proteases (thermolysin^62^ and carboxypeptidase-A^58,63^), named “anhydride pathway”, also called “glutamate-mediated nucleophilic pathway”. In this pathway, the sidechain carboxylate of the conserved active-site Glu residue (Glu140 in RVV-X numbering) directly attacks the scissile carbonyl carbon, producing an anhydride acyl-enzyme intermediate. Then, this intermediate is hydrolyzed by an incoming water molecule to release the products (Supporting Information, Scheme S1).^58,62^ However, X-ray diffraction and ^18^O isotopic labeling^58,64^ indicated that the Glu-mediated nucleophilic attack might only be viable for the hydrolysis of ester substrates but not for peptides.^59^ This is thought to be due to a misalignment of the reactant residues and the existence of an H-bond between the Glu and the substrate amide, which reduces the Glu nucleophilicity.^58^ Thus, the water-promoted mechanism for peptide substrates should be structurally and kinetically favored over the anhydride mechanism in RVV-X.^58,59^ Nevertheless, we have analyzed both mechanistic scenarios in this work.

In summary, the atomic-level details of the SVMP mechanism are still controversial, mainly because earlier studies relied on different types of metalloproteinases, and the reactions were studied with different substrates.^55−59,62^ Additionally, no studies have been made on snake venom orthologues. Therefore, a quantitative description of the free energy profiles and detailed atomic-level mechanisms is needed for the SVMPs, particularly given its central role in the snakebite envenoming pathophysiology. These facts together demand an urgent need for a deep understanding of the chemical transformations in the RVV-X active site. This is essential for designing new and more efficient tools, that is, readily accessible and affordable-for-all small-molecule inhibitors for early treatment of snakebite envenoming.

In this study, computational DFT:MM calculations were used to determine the reaction mechanism of RVV-X. We present the RVV-X proteolytic mechanism against FX at the DFT:MM level of theory, with atomic detail, including the geometry of all minima and transition states and the reaction free energy profile. In addition, the crucial role played by the Zn^2+^ cofactor in catalysis has also been elucidated.

As referred before, the metalloproteinases are highly conserved proteolytic enzymes. The catalytic sites of the SVMP members are structurally very similar, and all share the consensus signature sequence. SVMP structures have a typical topology, consisting of six α-helices and five stranded β-sheets in the M domain where the zinc ion is localized. Besides, sequence comparison between the M domains of SVMPs reveals quite high identity values of around 66%.^65^ This chemical and stereochemical match, particularly at the active site region, allows us to hypothesize that many of the members of this family of enzymes share a common catalytic mechanism. Therefore, our results concerning the intricacies of the RVV-X mechanism might be extrapolated to many other members of this vast family.

## Computational Methods

### Homology Modeling

Structural information on snake venom proteins (toxins) is limited and, therefore the structure of our target, RVV-X from *D. russelii*, is not available in the protein data bank. However, the structure of the Eastern RV (*Daboia siamensis*) RVV-X has been determined (PDB ID: 2E3X).^40^ The taxonomic proximity between the two vipers is so significant that some researchers still consider the latter a subspecies of the former. Consistently, the sequence identity between the enzymes of both species is nearly complete: the Eastern RV RVV-X structure showed 97.7% of sequence identity to RV HC and 100 and 91.0% to LC1 and LC2, respectively. This structure was co-crystallized with a clinical inhibitor, Ilomastat, with a resolution of 2.91 Å. Thus, a three-dimensional (3D) model of *D. russelii* RVV-X was trivially built, resorting to a homology modeling technique using the SWISS-MODEL web server,^66,67^ using the crystallographic structure from *D. Siamensis* RVV-X as a template. A list with the mutations and respective positions required to modify the RVV-X primary structure of *D. siamensis* to *D. russelii* is provided in the Supporting Information.

The superimposition of the backbone of both the Eastern’s RV template and RV target revealed a remarkable similarity, with an RMSD difference of 0.1 Å, which was expected due to the very high sequence identity. The HC was organized in four long α-helices, one of which contains the active site and a four-stranded β-sheet without the antiparallel β-sheet (β4). The catalytic zinc ion made a tetrahedral coordination sphere with His139, His143, His149, and the added water molecule (SI, Figure S2). The metalloproteinase domain of this structure was solvated and used for the calculations. The protonation states of titratable residues were predicted by the H++ web server (http://biophysics.cs.vt.edu/H++)^68,69^ and are presented in Supporting Table S1. In summary, all residues were predicted to be in their typical protonation states except for Glu90 and Glu140, which were predicted to be neutral at physiological pH. The first is exposed to the solvent and does not establish strong interactions or salt bridges that justify the neutral form; thus, we decided to model it in the anionic form. The second, Glu140, was interestingly predicted to have a p*K*_a_ slightly above 7.4, emphasizing its considerable basicity that is materialized through the barrierless and spontaneous abstraction of the nucleophilic water just at the beginning of the reaction, as will be seen below.

### Parametrization of the Metalloproteinase’s Metal Center

The python-based Metal Center Parameter Builder (MCPB.py)^70^ module of the Assisted Model Building with Energy Refinement (AMBER) Tools18 package was used to parametrize the metalloproteinase’s metal center (details in the SI).

To carry out classical molecular dynamics simulations of the RVV-X, the modeled protein was solvated in an octahedral box of TIP3P water molecules^71^ so that the box boundaries were at least 15 Å away from any protein atom. Finally, eight Cl^–^ counterions were added to achieve electroneutrality on the modeled system. Missing hydrogen atoms were added by the tLeap software from the AMBER18 package. The geometry of the entire system was minimized through four sequential steps using the AMBER18 package and the GAFF^72^ and ff14SB^73^ force fields (details in the SI). Considering that the chemical reaction occurs in the metalloproteinase domain, which is located in the HC, the studies focused only on its HC, i.e., the LC (snaclecs) were erased for the subsequent simulations.

### Protein–Peptide Docking

As the activation peptide structure is undetermined and disordered,^74^ it had to be modeled before the docking calculation. Due to its disordered nature, only an eight-residue peptide was used to model the cleavage region of the substrate, which is the portion that fits into the RVV-X binding site. To this end, the last 4 residues of the activation peptide (Asn-Leu-Thr-Arg) and the first 4 residues of the FXa serine protease N-terminal domain (Ile-Val-Gly-Gly) were modeled with tLeap. The peptide minimizations were carried out according to the same procedure used for the RVV-X model. With the substrate and target minimized, the next step consisted of protein–peptide docking. The latter was performed with the HPepDock (Hierarchical Flexible Peptide Docking)^75^ online server. A set of restraints was defined: the receptor residues (His143, Zn^2+^, Glu140) had to interact with the ligand residues (Thr3, Arg4, and Ile5) with a minimum and maximum distance of 2 and 6 Å. Finally, the most favorable solution was selected and further minimized (details in the SI). The final structure formed the obligatory interactions for the reaction: The scissile bond carbonyl coordinated to the Zn^2+^ cofactor, and the scissile peptide amine lay close to the Glu140 sidechain to allow for the amine protonation by Glu140. These observations further confirm that the position of the substrate is correct. In addition, the C-terminal portion of the scissile peptide segment (Val-Gly-Gly-Ile) adopted the same conformation as the peptide-like inhibitor GM6 co-crystallized with *D. siamensis* RVV-X^40^ (SI, Figure S3). A water molecule was introduced to complete the tetrahedral shell of the Zn^2+^ cofactor. It lay in the same position as the N3 atom of the GM6 inhibitor. Altogether, these observations showed that a correct geometry for the Michaelis complex was achieved.

The substrate was subsequently parameterized using AMBER’s ff14SB force field. We relaxed the modeled structure with a 170 ns molecular dynamics (MD) simulation (details in the SI). The results have shown that the modeled and optimized structure did not deviate from the ones in the MD ensemble, apart from the sidechain rotamer of Arg219, which goes deeper into the pocket S1 in the MD simulation. In this scenario, we decided to use the modeled and optimized structure to keep it as close as possible to the X-ray structure. The latter represents an average over all proteins in the crystal and thus is the most important and informative of all structures. Our experience is that the x-ray structure can provide results similar to ensemble averages because it is an ensemble average itself.^76,77^

### QM/MM Model

**Figure 3:**
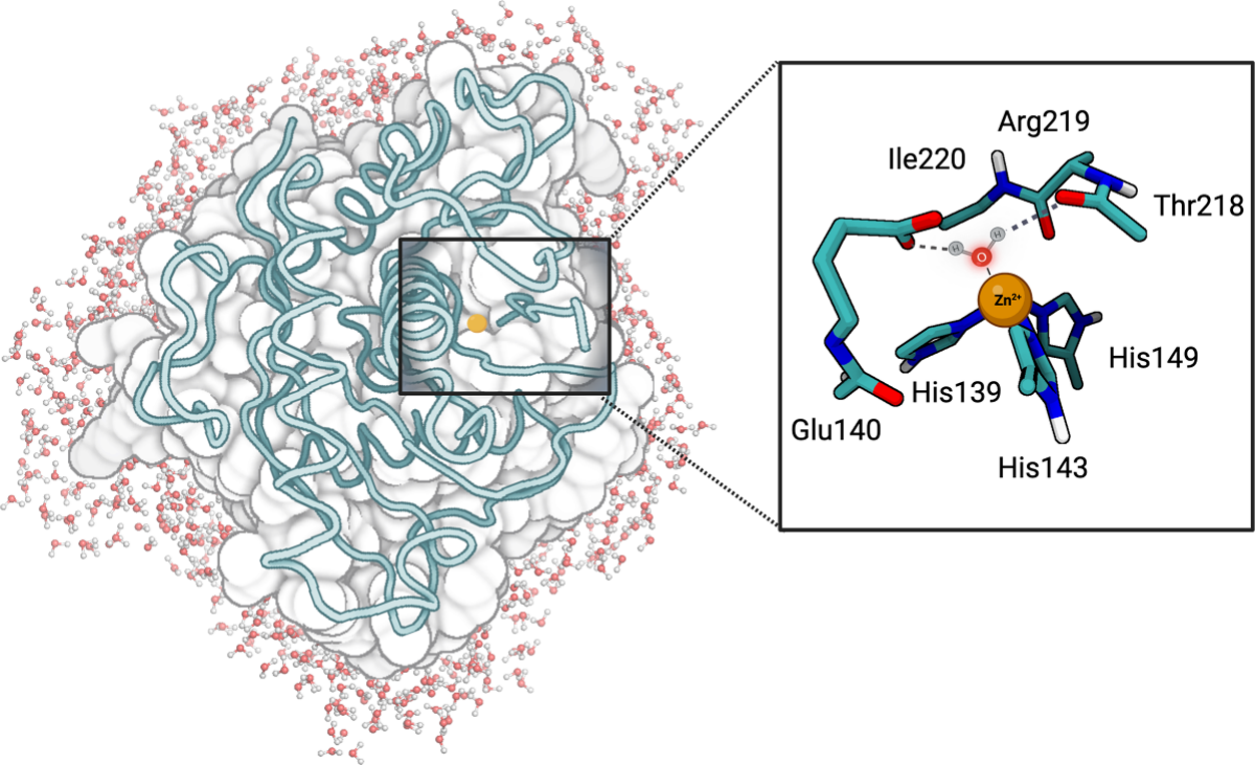
QM/MM model used in the calculations. (Left) The metalloproteinase domain (surface and ribbon), the solvent (CPK), and the cofactor (yellow sphere). (Right) Close-up view of the QM layer. Carbon is shown in green, nitrogen in blue, oxygen in red, hydrogen in white, and zinc in copper.

The minimized structure of the protein-substrate complex was loaded on the molUP plugin^78^ of the VMD program package, which provides a full-featured graphical user interface (GUI) to the software Gaussian.^79^ Only the metalloproteinase domain and the substrate were included in the model to reduce the computational burden. To simulate the aqueous environment, 3489 water molecules and one chloride ion were added, corresponding to a solvent shell thickness of 6 Å around the protein. The whole system consisted of 6954 atoms. Two ONIOM layers were defined within the “our own n-layered Integrated molecular Orbital and Molecular mechanics” (ONIOM) formalism: the quantum mechanics (QM) layer, described with density functional theory (DFT), and the molecular mechanics (MM) layer, described by the AMBER force field. The QM region included all of the atoms that undergo bond formation or breaking processes and those which made first-shell interactions with the reacting atoms or whose hybridization changed during the reaction:^80−82^ the Zn^2+^ ion, the catalytic water molecule, the side chains of His139, His143, and His149 until the β-carbon and the Glu140 residue, the substrate’s Arg219 backbone up to the β -carbon, the Ile220- α- and β-carbon plus the amine part, and the Thr218 α-carbon. This layer contained 73 atoms and a net charge of +1 (Figure 3). As typical with metalloenzymes, a relatively small QM layer encompassed all atoms relevant for the reaction. The QM part was described at the DFT level with the B3LYP hybrid functional,^83^ in combination with the 6-31G(d) basis set^84^ for geometry optimizations (larger basis sets were used for single-point energy calculations). The MM part included the rest of the protein and the solvent. It was treated with the AMBER ff14SB force field. Incomplete valences at the boundary between the two layers were completed with link hydrogen atoms.^80,85,86^

### Determination of the Stationary Points

Linear transit scans were initially performed to study the energy profile of the reaction’s steps and provide good guesses for the unrestrained optimization of minima and transition states. These were subsequently fully optimized (apart from the frozen atoms), their vibrational frequencies were calculated to confirm that the structures were indeed minima or transition states (i.e., having none or a single imaginary frequency, respectively), and IRC calculations^87^ were performed to confirm that the transition states were indeed the ones of interest. Furthermore, Grimme’s D3(BJ) dispersion corrections were added. In addition, the zero-point (ZPE), thermal energy, and rigid rotor/harmonic oscillator entropy were calculated at 298.15 K and 1.0 bar. Finally, single-point energy calculations at the ONIOM (B3LYP/6-311++G(2d,2p):AMBER), ONIOM(B3LYP/6-311++G(3df,2pd):AMBER), ONIOM(M06/6-311++G(3df,2pd):AMBER) and ONIOM(M06L/6-311++G(3df,2pd):AMBER) levels of theory were carried out.

Additionally, a set of different initial structures was retrieved from a 100 ns MD simulation (details in the SI) in the NPT ensemble with the same setup as the ones explained above, to evaluate the influence of the starting conformation on the free energy barrier of the rate-limiting step. Ten distinct structures that possessed suitable interatomic distances for the catalysis to occur were then selected. Following optimization of each retrieved structure, potential energy surface scans were performed using the B3LYP/6-31G(d) level of theory. Zero-point (ZPE), thermal energy, and rigid rotor/harmonic oscillator entropy were calculated and single-point energy calculations at the B3LYP/6-311++G(3df,2pd) were used to determine the energetic barrier for each conformation. Grimme’s D3(BJ) dispersion corrections were further added.

Images were created with PyMol and BioRender.com.

## Results and Discussion

### Michaelis Complex

**Figure 4:**
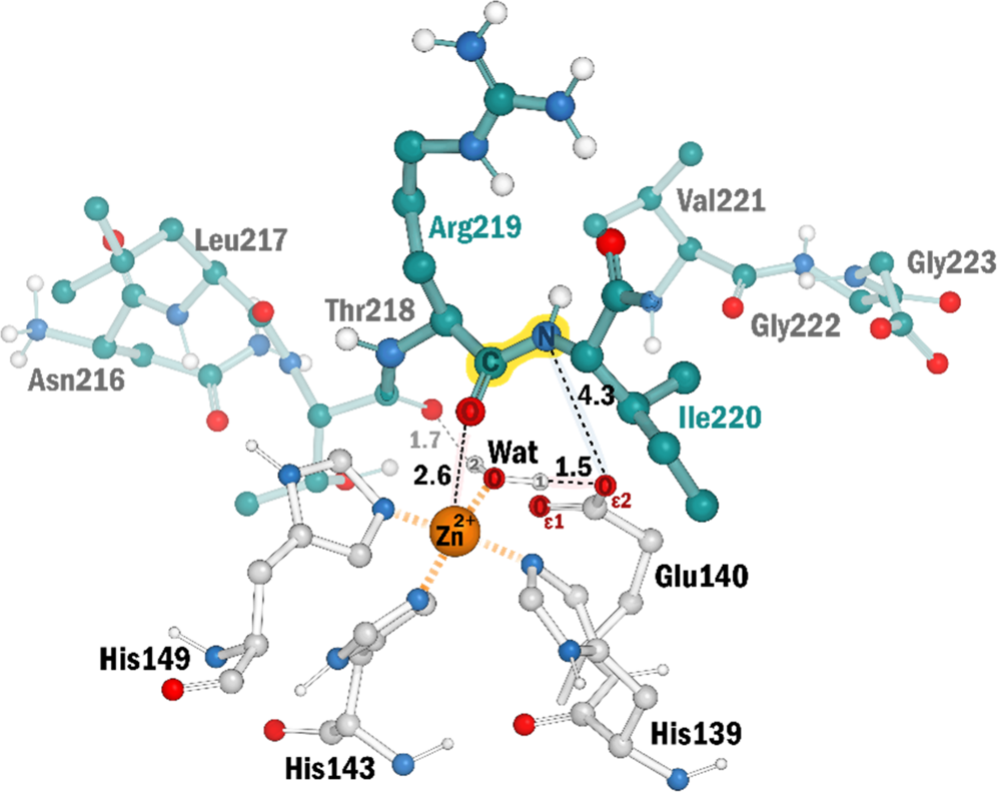
Michaelis complex as predicted by restrained docking and modeling, in which the carbonyl oxygen (O_pep_) of the scissile peptide bond (Arg219-Ile220), the catalytic water (O_wat_), and the three histidine residues (His139, His143, His149) are coordinated to the Zn ion. Distances between the catalytic residues are presented. The yellow shadow highlights the peptide bond being broken.

In the structure of the protein–peptide complex obtained from the docking of the eight-segment peptide in the RVV-X binding site (SI, Figure S4A,B), the metal ion has a distorted tetrahedral coordination sphere, with the three histidine residues of the catalytic motif (SI, Figure S4A), the catalytic water, and the substrate’s carbonyl oxygen atom bound to it, with distances ranging from 1.89 to 2.62 Å. The zinc ion is well coordinated, and the backbone of the scissile residues—Arg219 and Ile220—is correctly oriented for the cleavage process. The Zn^2+^-coordinated water molecule is hydrogen-bonded to Glu140 (1.5 Å) and close to the Arg219 carbonyl carbon atom. In summary, the Michaelis complex structure is suitable for the reaction to proceed through the “water-promoted pathway” mechanism (Figure 4).

Moreover, with a distance of 2.85 Å, the zinc-bound water nucleophile is ideally positioned to attack the scissile peptide bond’s carbonyl carbon (C_pep_). This rearrangement is sustained by two hydrogen bonds given by the Glu140 sidechain (H1_wat_-Oε2_Glu_) and the Thr218 (H2_wat_-O_Thr_) of the peptide substrate, with distances of 1.53 and 1.66 Å. During the subsequent QM/MM geometry optimization, the metal ion maintained its five-coordination environment. However, O_pep_ moved slightly away from the Zn^2+^ ion (2.65 Å), and the water molecule got closer to the Zn^2+^ ion (Zn^2+^–O_wat_ distance of 1.90 Å). One of the water protons was immediately transferred to Glu140 (Oε2_Glu_-H1_wat_ bond length of 1.07 Å), with O_wat_ keeping a hydrogen bond with the transferred proton (1.44 Å). The nucleophilic water molecule is very polarized due to the interactions with the negatively charged carboxyl group of Glu140 base and the acidic Zn^2+^ ion. In these conditions, the p*K*_a_ of the water molecule drops considerably,^88,89^ and in this case, it dropped enough to allow for the barrierless deprotonation by Glu140. Thus, the nucleophile for the water-mediated pathway, i.e., the Zn^2+^-bound hydroxide ion was swiftly generated.

On the other hand, the RVV-X structure indicates that the anhydride catalytic pathway is unlikely because the glutamate residue and the substrate’s carbonyl group are not in a favorable position for the reaction to take place with low barriers. The distance between the Glu140 sidechain oxygen atom closest to the carbonyl carbon of the scissile bond is 4.28 Å. Given the difficulty in dragging the enzyme backbone to approach the tightly coordinated substrate, the free energy barriers for navigating through this mechanism might be significant. Moreover, the nucleophilicity of Glu140 is severely compromised by the water molecule’s spontaneous and barrierless protonation.

**Figure 5:**
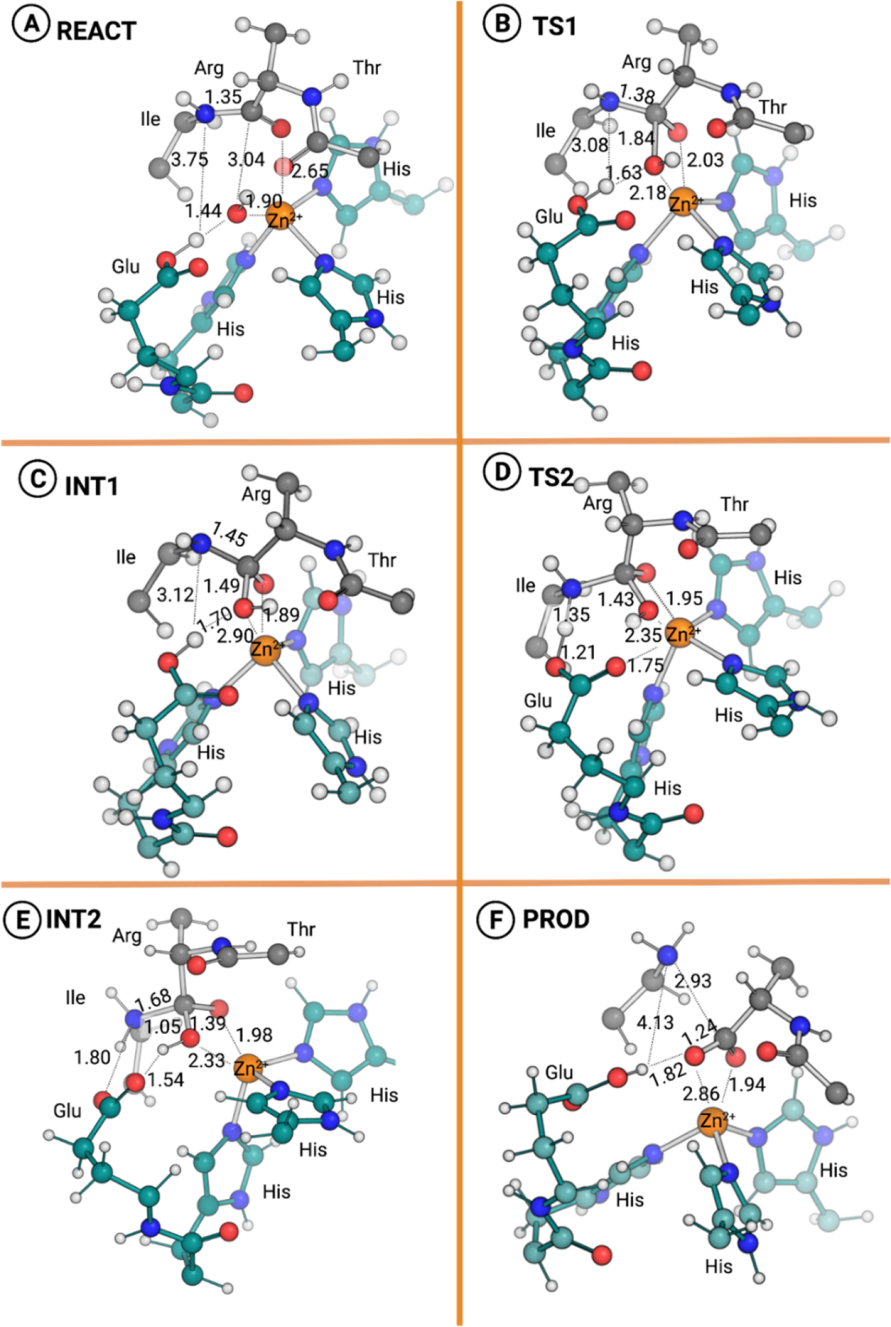
Fully optimized stationary states along the catalytic cycle of RV RVV-X. Only the QM layer is shown; the MM layer has been deleted for clarity. (A) Enzyme–substrate complex, (REACT); (B) first transition state, TS1, corresponding to the nucleophilic attack of the Zn^2+^-bound hydroxide ion to the peptide carbonyl carbon; (C) first intermediate (INT1), which corresponds to a Zn^2+^-bound doubly coordinated gem-diolate; (D) second, rate-limiting transition state (TS2) resulting from a proton transfer from Glu140 to the amine nitrogen of the scissile bond and partial cleavage of the C_pep_-N_pep_ peptide bond; (E) second intermediate, INT2, with the peptide amine nitrogen protonated and peptide bond severely weakened but not broken; (F) product, PROD, with the peptide bond fully broken and the product formed. To complete the cycle, a bulk solvent water molecule enters the binding site, coordinates to the Zn^2+^ ion, and displaces the C-terminal fraction of the product from the cofactor and active site. The most relevant catalytic distances are presented in Ångström.

In summary, an enzyme–substrate complex (REACT) is formed in which the hydroxide ion is ready to attack the scissile peptide bond (Figure 5A). This Michaelis complex differs from the proposals for the human matrix metalloproteinases; it is much more reactive and prone to carry out the water-promoted pathway.

### Revealing the Catalytic Mechanism of RVV-X

#### Glutamate-Assisted Nucleophilic Pathway

Even though the active site-substrate architecture naturally favors the water-promoted pathway, we performed a linear transit scan along the distance between the Glu140 Oε_2_ atom and the Cpep. As expected, the Glu140 nucleophilic attack led to a steep rise in energy up to prohibitive values. The Glu140 has an unproductive position for the attack as it is far from Cpep (5.1 Å), and thus the approximation implies high reorganization energy. In addition, it is a poor nucleophile due to the water molecule’s Zn^2+^-promoted spontaneous and barrierless protonation. In line with all previous observations here, the “Glutamate-assisted nucleophilic pathway” cannot explain the reactivity of RVV-X. As this pathway led to a very high energy rise for the first reaction step and a stable intermediate was not formed, we considered this mechanism unfeasible and continued by studying the water-promoted pathway.

#### Water-Promoted Pathway, First Step—Nucleophilic Attack

The first step of the proposed reaction is the deprotonation of the Zn^2+^-bound water molecule by Glu140. However, this step is barrierless at the active site of RVV-X, and thus the Michaelis complex already has the hydroxide ion formed at the active site. Thus, the first step of the mechanism of RVV-X is the nucleophilic attack of the Zn^2+^-bound hydroxide ion on the carbonyl carbon of the substrate’s scissile bond. The O_wat_–C_pep_ distance was chosen as the reaction coordinate. This step resulted in the formation of the first transition state (TS1, Figure 5B) and the first intermediate (INT1, Figure 5C). These stationary states were subsequently entirely and unconstrainedly optimized (after IRC calculations for the two minima). At TS1, the O_wat_–C_pep_ distance shortened to 1.84 Å, and the Zn^2+^–OH^–^ distance increased to 2.18 Å. Simultaneously, the distance between O_pep_ and Zn^2+^ also decreased from 2.65 to 2.03 Å, which is expectable as O_pep_ receives the negative charge of the hydroxide and becomes anionic, along with the change of hybridization of C_pep_ to sp^3^. This first step yielded a free energy barrier of 17.7 kcal·mol^–1^. The vibrational frequency involving the reaction coordinate for TS1 resulted in a single imaginary value of 218i cm^–1^. Furthermore, the distance between the Glu140 proton and the nitrogen atom of the scissile peptide bond (N_pep_) decreased from 3.75 to 3.08 Å when moving from the reactant to TS1. In the first intermediate (INT1, Figure 5C), the O_wat_–C_pep_ distance is 1.49 Å, and O_pep_ gains an sp^3^ hybridization and becomes negatively charged.

### Second Step—Proton Transfer and Peptide Bond Cleavage

In the second step, the sidechain of Glu140, which acts as a proton shuttle, donates the proton taken from the catalytic water to the scissile-bond nitrogen (N_pep_), triggering the breaking of the peptide bond. Thus, the distance between the Glu140 hydrogen and the scissile-bond nitrogen (H_Glu_–N_pep_) was chosen as the reaction coordinate, whose scan led to the identification of the second transition state (TS2, Figure 5D), which was subsequently fully and freely optimized. The product of this step and the second intermediate of the cycle (INT2, Figure 5E) was determined through IRC calculations and subsequently entirely and unconstrained optimized. At the TS2, the proton taken from the catalytic water molecule by the Glu140 was transferred to N_pep_, leading to a sharp drop in the H_Glu_–N_pep_ distance, from 3.12 Å at INT1 to 1.35 Å at TS2. Furthermore, the C_pep_–N_pep_ peptide bond distance increased from 1.45 at INT1 to 1.57 Å at TS2 (SI, Figure S5).

The hydrogen-bond network is rearranged along this step: the hydrogen bond between the peptide’s hydroxyl group of the scissile bond and the backbone oxygen of Thr218 (OH_pep_–O_Thr_) is replaced by a new H-bond between the protonated carboxyl group of the peptide’s scissile bond and the Oε1 of Glu140 (OH_pep_–Oε1_Glu_), with a distance of 1.75 Å (SI, Figure S6). This spontaneous H-bond rearrangement greatly facilitates the protonation of Glu140 by the substrate’s C-terminal carboxylic acid, which is very acidic at this stage due to the p*K*_a_ drop induced by its coordination to the Zn^2+^ cofactor. Moreover, the proton of the Glu140 sidechain carboxylate is no longer bonded to the carboxyl group but is instead halfway between the carboxylate and the peptide bond nitrogen. These events led to forming a Zn^2+^-bound gem-diolate transition state with both oxygens of the C-terminal carboxylic acid coordinated to the metal ion in a bidentate fashion with distances of 2.35 and 1.95 Å, the shorter distance corresponding to the anionic oxygen. The free energy barrier for this step is the highest of the cycle, 23.1 kcal·mol^–1^ above the reactants, becoming the rate-limiting transition state. The vibrational frequency for the TS2 structure is 1046*i* cm^–1^.

Interestingly, this coordination mode is mimicked by hydroxamate-based inhibitors such as batimastat or marimastat, which were developed as antineoplastic drug candidates targeting the human matrix metalloproteinases but failed at clinical trials. These drugs are under investigation to treat snakebite, as they are highly efficient in inhibiting the SVMPs in vitro and in vivo.^14,20,90^ The drug candidate’s carbonyl group resembles the substrate’s carbonyl group, and the hydroxamic acid hydroxyl group resembles the hydroxide ion (TS2, Figure 5D and SI, Figure S3). The fact that these drug candidates precisely mimic the rate-limiting transition state of the whole cycle reinforces the strategy of inspiring drug discovery on the rate-limiting transition states determined through accurate QM/MM calculations.

The rearrangements resulted in a gem-diolate intermediate INT2 (Figure 5E) with a severely weakened C_pep_–N_pep_ bond (1.68 Å). At this stage, the Glu140 proton has been transferred entirely to N_pep_. Simultaneously, there was a decrease in the distance between the Zn^2+^ and the substrate hydroxyl group (2.33 Å) (SI, Figure S5). The Zn^2+^–Opep distance increased to 1.95 Å as it moved from an oxyanion (C–O^–^) to neutral oxygen (C=O). The INT2 structure retained the distorted penta-coordinated shell around the Zn^2+^ cofactor (Figure 5E), composed of the oxygen atoms of the C-product carboxyl group (2.33 and 1.98 Å) and the histidines 139, 143, and 149.

**Figure 6:**
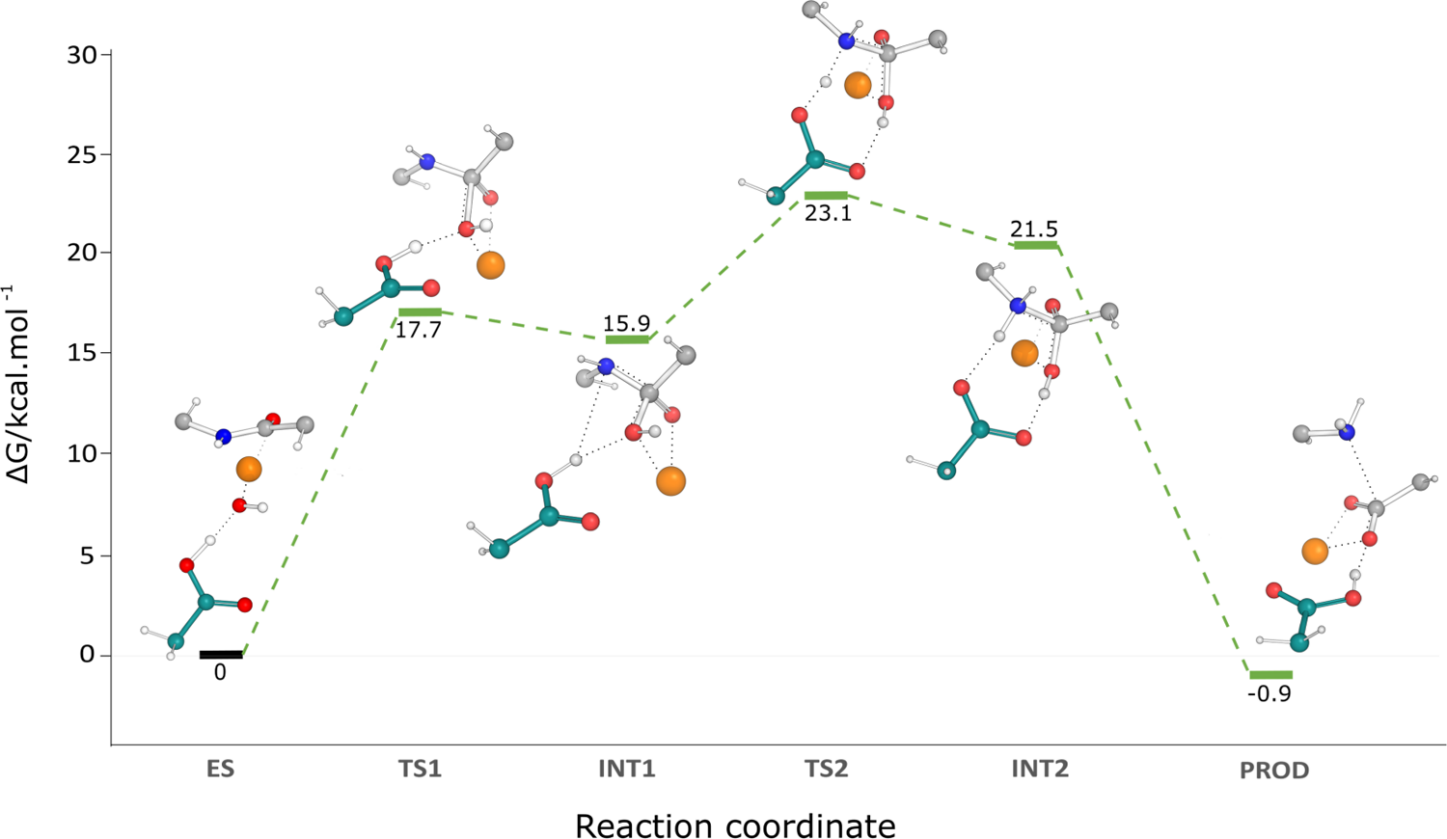
Water-promoted pathway: Gibbs energy profile along the reaction progress at the B3LYP/6-311++G(3df,2pd) with added Grimme’s D3-BJ dispersion corrections. The critical residues for each reaction step are shown in CPK representation. The first and second steps of the reaction yielded activation free energies of 17.7 and 23.1 kcal·mol^–1^, respectively. The reaction is exergonic; the overall reaction free energy is −0.9 kcal·mol^–1^.

As the peptide bond was not fully broken at the end of the second step, the C_pep_–N_pep_ distance was used as the reaction coordinate for the last step. The energy barrier of the definitive breaking of the peptide bond was less than 1 kcal·mol^–1^. The transition state between the INT2 and the products, with the peptide bond completely broken (PROD, Figure 5F), was not further optimized because the slope of the potential energy surface in the respective region was very flat. The associated barrier is very shallow (close to *k*_B_*T*)^91^ at the biologically relevant range of temperatures (25–37 °C), i.e., the range of body temperatures of the principal RV prey, for example, rodents. Therefore, the crossing rate from INT2 to TS3 is within the same timescale as bond vibration. This very fast step results in the formation of PROD. This reaction is exergonic, with a sharp free energy drop at PROD to −0.9 kcal·mol^–1^ (Figure 6), resulting from the peptide bond’s cleavage and leading to a C_pep_–N_pep_ distance of 2.93 Å. At the end of this step, the proton of the substrate’s carboxyl group and the proton of the Oε2Glu are entirely transferred to the Glu140 and the amine-free nitrogen, respectively. The Zn^2+^–O_wat_ distance increases from 2.33 to 2.86 Å, which leads to a short decrease in the Zn^2+^–O_pep_ distance (SI, Figure S5). These events determine the end of the reactive part of the enzyme cycle.

Albeit the product is formed, the enzyme is not entirely reconstituted. The reactant state’s complete regeneration still involves replacing the bound product with a water molecule from the bulk solvent; the carboxylate of the leaving C-terminal portion of the product probably deprotonates Glu140 and fully regenerates the enzyme for a new cycle. However, the product’s release is very challenging to simulate, and as it is a physical process that is well understood but demands extensive sampling to simulate, it is generally not included in mechanistic studies of enzyme catalysis. Figure 6 shows the free energy profile for the complete reaction and the evolution of the critical distances along the complete reaction cycle is presented in the SI, Figure S5. See Supporting information, Scheme S2 for a schematic diagram of the final reaction mechanism for the RVV-X.

The rate-limiting step (23.1 kcal·mol^–1^) is higher than the one in experimental studies for MMP-2, 15–17 kcal·mol^–1^, obtained by kinetic studies.^56,92,93^ However, these experimental studies have been made with different enzymes, hindering the direct comparison of values. Zinc hydrolases generally have rate-limiting free energies of 16.9 ± 2.1 (mean ± SD)^94^ The value found here is consistent with this interval, and the difference is within the error of the computational method (3–5 kcal·mol^–1^). In addition, it is well known that the accurate description of energy barriers demands a significant fraction of HF exchange, but the description of transition metals demands a low fraction of HF exchange or no HF exchange. Therefore, there is a conflict in the need for HF exchange in studying barriers involving transition metals, such as the case here. In the face of this, and to evaluate the influence of the density functional in the results, we recalculated the ONIOM energy with the M06 and M06-L density functionals and the 6-311++G (3df, 2pd) basis set (results in Table S2). The results were very similar with all functionals, with an MUE between B3LYP and M06 of 2.4 kcal·mol^–1^ and between B3LYP and M06-L of 0.8 kcal·mol^–1^. The rate-limiting step with M06 and M06-L was lower, 17.8 and 21.1 kcal·mol^–1^, respectively.

The potential energy surface of 10 additional starting structures taken from a 100 ns MD simulation was analyzed, from which the lowest and highest obtained free barriers (TS2 in relation to ES) ranged from 15.85 to 26.98 kcal·mol^–1^ (Table 1), at the B3LYP/6-311++G(3df,2pd)-D3:ff14SB level of theory. Many of these free energy barriers ranged from 18 to 22 kcal·mol^–1^. Although the 10 starting structures exhibited a quite similar QM region with almost the same interatomic distances, these nonetheless account for nearly 11 kcal·mol^–1^ energy difference. No pronounced differences in the high-level region of the structures were found that could justify the variation of the above-mentioned barrier, meaning that the differences come from medium- and long-range interactions. In other studies of enzyme reaction mechanisms, a significant spread in the activation energy values originated from different starting structures has also been observed, with a low-tail close to the experimental value and a spread to larger values corresponding to enzyme structures that are not perfectly pre-organized to react.^95−97^ Here, the scenario is not different. Finally, as the observed rate is exponentially dominated by the lowest barriers,^98^ the calculations further confirm that the rate-limiting step is close to the experimental studies for MMP-2, 15–17 kcal·mol^–1^, as previously mentioned.

**Table 1:** Rate-limiting Activation Free Energies at the ONION(B3LYP/6-311++G(3df,2dp)-D3:MM) Level Starting from 10 Initial Structures

conformation	rate-limiting Δ*G*_act_/kcal·mol^–1^	conformation	rate-limiting Δ*G*_act_/kcal·mol^–1^
X-ray	23.1	conf. 6	27.0
conf. 1	21.5	conf. 7	24.4
conf. 2	17.7	conf. 8	22.8
conf. 3	21.4	conf. 9	22.2
conf. 4	19.7	conf. 10	15.8
conf. 5	17.0		

### Role of the Zn^2+^ Cofactor

The Zn^2+^ cofactor has many different roles. It is obviously important for coordinating the substrate and the organization of the active site. Nevertheless, we wanted to understand its role in the first-order reaction rate, i.e., in lowering the activation free energy of the chemical steps, with an emphasis on the rate-limiting step. To do so, we calculated the contribution of the Zn^2+^ ion to the barrier of each reaction step (SI, Figure S7). For that purpose, we subtracted the Zn^2+^ ion energy and interactions (i.e., recalculating the energy in single-point calculations with the 6-31G(d) basis set but deleting the Zn^2+^ ion) and only the electronic energy of the QM layer was used. When comparing the energy profile without the Zn^2+^ cofactor with that obtained for the wild-type using the same basis set and including only the QM layer, the rate-limiting barrier for the wild-type reaction was 13 kcal·mol^–1^ lower than the value obtained without the cofactor (22.9 kcal·mol^–1^).

Without the cofactor, the REACT state is not the ground state anymore. Instead, the ground state has an anionic Glu140 and a neutral water molecule, as the Glu140 does not spontaneously and barrierlessly deprotonate the nucleophilic water molecule. Without the cofactor, the rate-limiting step of the whole cycle becomes precisely the deprotonation of the nucleophilic water, and the rate-limiting barrier increases by 9.0 kcal·mol^–1^. Thus, it becomes clear that the principal role of the metal ion is the in situ generation of the hydroxide nucleophile.

The problem of generating a strong nucleophile at the active site is transversal for the protease superfamily.^99^ The nucleophile has to be generated in situ to avoid side reactions. For example, most serine proteases rely on a Ser–His–Asp/Glu triad to overcome the burden of deprotonating the very basic serine residue, whose p*K*_a_ in aqueous solution is very high.^100^ At the active site, the slightly basic histidine residue efficiently deprotonates the serine because the Asp/Glu residue of the triad is pre-organized to stabilize the positive form of the histidine that results from the serine deprotonation. The same principles and catalytic machinery can be found in the large family of cysteine proteases.^101^ In both families, the oxyanion generated upon nucleophilic attack is generally stabilized by an oxyanion hole constituted by two hydrogen-bond donors, often backbone amines.^99−101^

In the case of aspartic proteases, the existence of a pair of active-site Asp residues, one neutral and one negative, allows overcoming the burden of deprotonating the very basic water molecule. While one of the Asp residues deprotonates the hydrolytic water molecule, the second Asp residue protonates the very basic aliphatic oxyanion that is generated by the water attack on the substrate’s carbonyl^98^ so the burden of the deprotonation is compensated by the simultaneous very exothermic protonation of the growing oxyanion.^99,102^ In some cases, the protonation and deprotonation can be carried out by a single Asp residue.^103^

The strategy of RVV-X for generating the nucleophile is to coordinate the water molecule to the Zn^2+^ cofactor, lowering the water p*K*_a_ through the much stronger coordination of the hydroxide ion in relation to the neutral water. The water deprotonation molecule moves the negative charge from the non-Zn^2+^-coordinated Glu140 to the Zn^2+^-coordinated water molecule, with an obvious electrostatic gain. In the subsequent reactions, the number of ionic oxygen atoms coordinated to the Zn^2+^ ion is kept unchanged—there are always two Zn^2+^-bound oxygen atoms, one negative and one neutral, except for the Michaelis complex before the water deprotonation, where both Zn^2+^-coordinated oxygen atoms are neutral. Thus, it makes sense that the cofactor does not make a decisive contribution to lowering the subsequent energy barriers.

Given the very significant sequence identity among zinc metalloproteases, particularly at the active site, it is tempting to speculate that the role of the Zn^2+^ cofactor might be transversal to the family of metalloproteases. However, such generalization, albeit logical and even probable, needs a solid analysis ground in further family members.

### Comparison with Other Studies

The obtained stationary geometries are close to those found in studies of human matrix metalloproteinases.^55,56,59,104^ The catalytic reaction follows a two-step mechanism consistent with the study carried out by Pelmenschikov and Siegbahn on human Matrix metalloprotease-3 (MMP-3).^55^ However, unlike the proposed mechanism for MMP-3, the rate-limiting step in the RVV-X mechanism is not the nucleophilic attack but the protonation of the leaving amino group. This last finding agrees with the study carried out by Díaz and Suárez on the human MMP-2 enzyme toward two peptide substrates.^56^

Overall, the QM/MM results provide solid support for the water-promoted pathway mechanism proposed for the human MMPs. Furthermore, we show that the pre-reactive complex is well organized for the reaction to occur according to this proposal. In 2015, Vasilevskaya and their colleagues demonstrated that pulling out the C-product from the active site (estimated free energy barrier of 20 kcal·mol^–1^) causes the product’s NH2 group to rotate, allowing the Glutamate carboxyl group also to rotate and, in turn, protonate the N-product.^104^ However, Pelmenschikov and Siegbahn showed in 2002 that the thermolysin N-product could be released in its neutral state without needing a second proton transfer.^62^

## Conclusions

We present the first simulation of the reaction mechanism of RVV-X, an SVMP. This toxin is of keen interest due to its pharmacological effects following envenomation and biotechnological applications. The findings of this study on the RVV-X mechanism are generally consistent with the proposed “water-promoted pathway” mechanism of human metalloproteinases. The most evident difference is that the RVV-X ground state already has the nucleophilic hydroxide ready for action. The latter is generated in situ through spontaneous and barrierless deprotonation due to the extensive lowering of the p*K*_a_ of the Zn^2+^-bound water molecule and the pre-organization of Glu140 in an ideal position for deprotonation. The results further indicate that the most significant role of the Zn^2+^ cofactor is precise in generating the nucleophile; its role in stabilizing the following transition states is relatively minor. This role is well framed within the challenges that the large and ubiquitous protease superfamily face to hydrolyze peptide bonds. The high active site conservation might be a general trait of zinc metalloproteinases. In addition, we show that the coordination mode of therapeutic inhibitors of the human MMPs, such as batimastat and marimastat, presently under study for snakebite treatment, perfectly mimics the one of the rate-limiting transition of RVV-X state found in this study. Thus, understanding the reaction mechanism and catalysis of this fantastic enzyme shall open avenues for the rational development of other high-affinity RVV-X inhibitors with relevant therapeutic potential to combat the RV envenomation.

## Data Availability

The cartesian coordinates and the force field parameters of all stationary points discussed in this paper are available in the Supporting Information as a ZIP file. The system was prepared using AMBER18 software. The AMBER package can be purchased at https://ambermd.org/index.php. H++ web server (http://biophysics.cs.vt.edu/H++) was used for the prediction of the protein titratable residues p*K*_a_. VMD v1.9.4 and PyMOL v2.3.0 were used for visualization and analysis of the system. VMD can be downloaded from https://www.ks.uiuc.edu/Research/vmd/ and PyMOL can be downloaded from https://pymol.org/. The VMD MolUP extension was used to prepare Gaussian input files and to visualize results from Gaussian output files. The molUP extension can be downloaded from https://biosim.pt/molup/. Gaussian 09 D01 was used for the QM/MM calculations. Gaussian software can be purchased from https://gaussian.com/.

## Supporting Information Available

SVMP different classes; model construction; p*K*_a_ values; minimization and molecular dynamics simulations; stationary points; energetic data; representation of the anhydride pathway, and representation of the final mechanism obtained for RVV-X (PDF)
PDB files and Gaussian input files used for the optimization of the stationary points, parameter files of the initial system, and restart file of the minimized structure (ZIP)
