# Supplementary material for: Unraveling the Reaction Mechanism of Russell’s Viper Venom Factor X Activator: A Paradigm for the Reactivity of Zinc Metalloproteinases?
Source: J Chem Inf Model. 2023 Apr 24;63(13):4056–69. doi: 10.1021/acs.jcim.2c01156 (PMC10336966; doi:10.1021/acs.jcim.2c01156)
Supplement: Supplementary file 1 — ci2c01156_si_001.pdf [file ci2c01156_si_001.pdf]

Supporting Information

Unraveling the reaction mechanism of Russell's viper venom factor X activator: a paradigm for the reactivity of zinc metalloproteinases?

J. Castro-Amorim[1], Ana Oliveira[1], Ashis K. Mukherjee[2], Maria J. Ramos[1], Pedro A. Fernandes[1,*]

[1]LAQV, REQUIMTE, Departamento de Química e Bioquímica, Faculdade de Ciências, Universidade do Porto, Rua do Campo Alegre, s/n, 4169-007 Porto, Portugal

[2]Institute of Advanced Study in Science and Technology, Vigyan Path Garchuk, Paschim Boragaon, Guwahati-781035, Assam, India

e-mail: pafernan@fc.up.pt

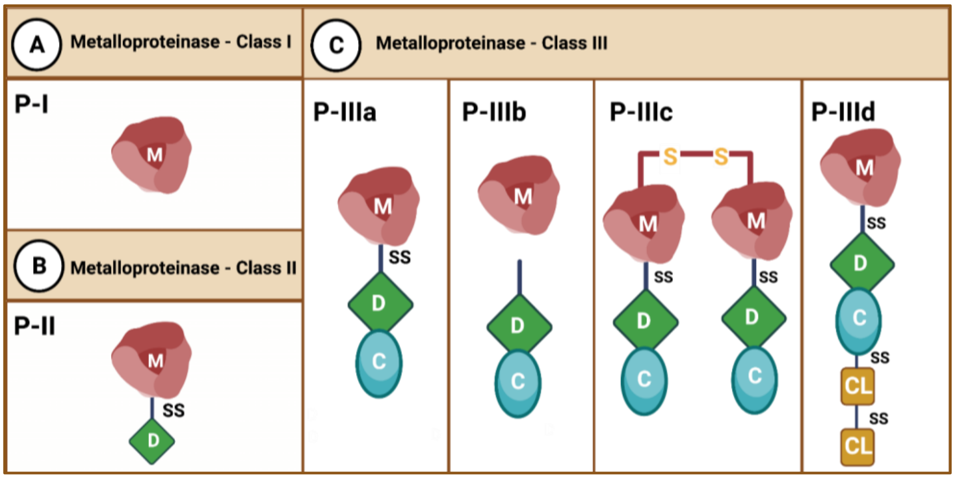

Figure S1: The three classes of SVMPs, P-I (A), P-II (B), and P-III, and the P-III subclasses a, b, c, and d (C). M: metalloproteinase domain; D: Disintegrin-like domain; C: Cys-rich domain; c-L: snake C-type lectin-like domain; P-III is subdivided into P-IIIa, the canonical structure, PIII-b which might result from proteolytic cleavage, P-IIIc that forms homo- or heterodimers linked by disulfide bridges and P-IIId that forms a complex with two covalently bridged snake C-type lectin-like proteins. Created with BioRender.com

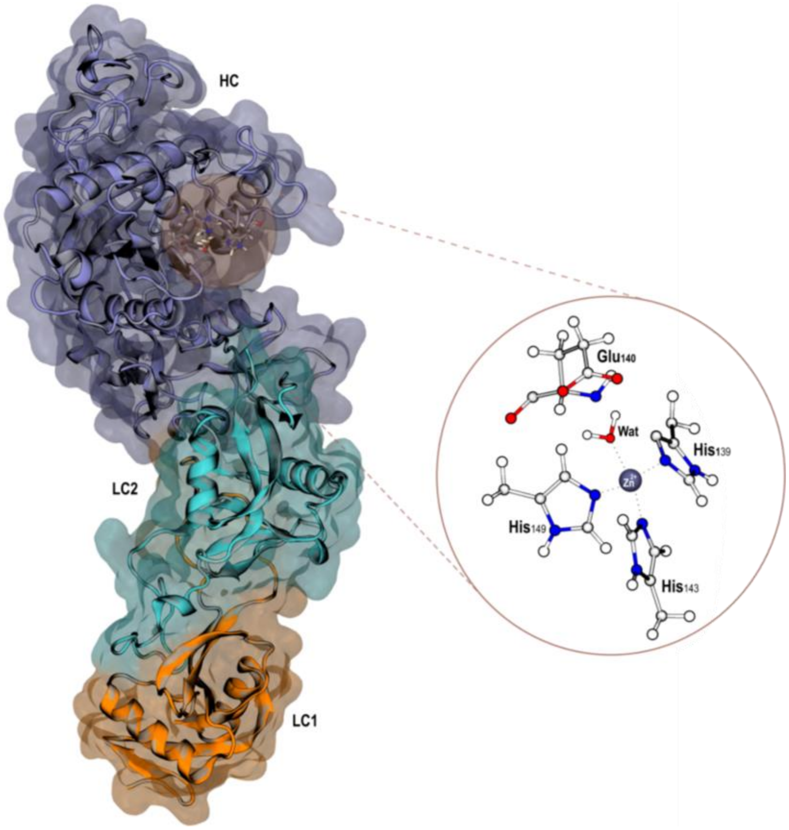

Figure S2: Structural superimposition of the target (colored by chain, HC: ice blue, LC1: orange, LC2: cyan) and the template (gray) and a close-up view of the model's catalytic center with the modeled catalytic water.

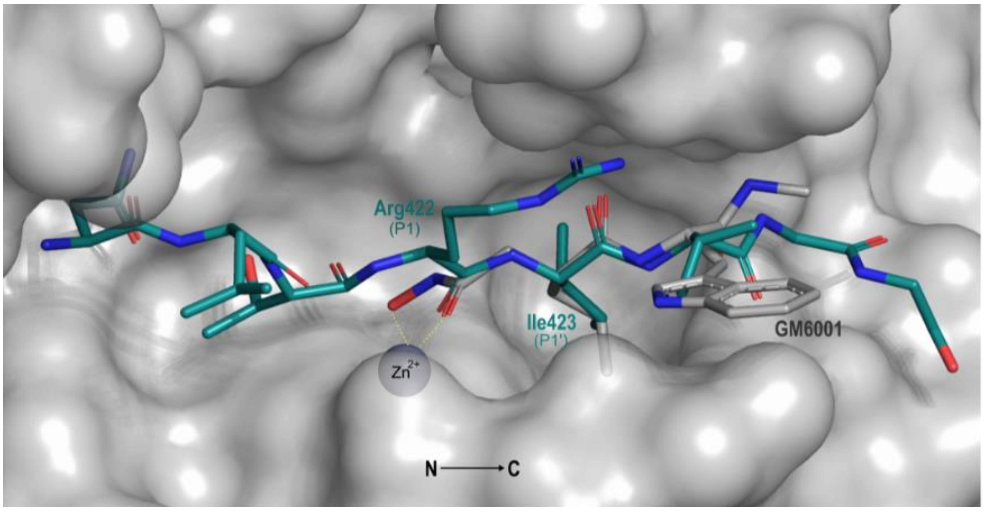

Figure S3: Close-up view of the docking model of RVV-X binding pocket with both the FX cleavage region and the inhibitor GM6001 superimposed. Both the inhibitor's and the substrate's carbonyl groups resemble one another. The residues of the peptide segment are shown in blue, and those of the inhibitor are shown in gray.

Table S1: pKa values of the protein titratable residues at pH=7 predicted by the H++ web server. (http://biophysics.cs.vt.edu/H++) [1, 2].

Residue	pKa	Residue	pKa	Residue	pKa	Residue	pKa	Residue	pKa
Asp 14	6.72	Lys 4	9.25	Arg 59	12.44	Glu 8	6.75	Tyr 31	10.00
Asp 58	3.91	Lys 19	10.51	Arg 79	9.80	Glu 32	4.36	Tyr 117	11.85
Asp 60	4.81	Lys 20	10.44	Arg 86	15.04	Glu 39	5.03	Tyr 148	10.39
Asp 69	4.17	Lys 29	9.72	Arg 99	12.32	Glu 54	2.78	Tyr 183	12.77
Asp 73	6.64	Lys 87	10.89	Arg 118	12.03	Glu 70	4.62	Tyr 186	13.10
Asp 82	3.52	Lys 132	11.13	Arg 129	12.44	Glu 77	6.17	Tyr 190	10.62
Asp 90	8.66	Lys 152	10.48	Arg 185	12.55	Glu 140	8.43	Tyr 197	9.65
Asp 97	4.56	Lys 173	10.45	Arg 189	12.66	Glu 213	5.82	Tyr 254	10.01
Asp 101	3.66	Lys 191	10.24	Arg 201	12.53	Glu 216	6.41	Tyr 303	9.76
Asp 150	4.78	Lys 193	9.85	Arg 266	12.75	Glu 217	4.61	Tyr 305	12.17
Asp 158	4.19	Lys 202	10.60	Arg 267	12.38	Glu 219	7.96	Tyr 340	10.31
Asp 169	2.61	Lys 240	10.42	Arg 269	13.41	Glu 220	3.94	Tyr 341	11.10
Asp 182	4.67	Lys 242	10.41	Arg 287	12.37	Glu 246	4.49	Tyr 343	9.71
Asp 203	4.22	Lys 257	10.57	Arg 301	12.47	Glu 271	3.58	Tyr 381	11.78
Asp 222	4.28	Lys 259	10.17	Arg 313	12.25	Glu 276	4.60	Tyr 416	12.69
Asp 228	4.01	Lys 328	10.41	Arg 323	12.36	Glu 284	4.40		
Asp 235	2.41	Lys 337	11.50	Arg 345	12.38	Glu 334	3.36		
Asp 273	3.63	Lys 346	10.99	Arg 350	12.41	Glu 347	4.55		
Asp 288	5.23	Lys 351	9.08	Arg 363	10.70	Glu 398	4.70		
Asp 308	5.23	Lys 360	9.93	Arg 372	12.46				
Asp 329	2.48	Lys 374	10.46	Arg 407	12.67				
Asp 358	4.52	Lys 388	9.13						
Asp 385	4.58	Lys 396	11.24						
Asp 392	5.66	Lys 401	10.73						
Asp 399	2.70	Lys 406	9.75						
Asp 411	3.45								

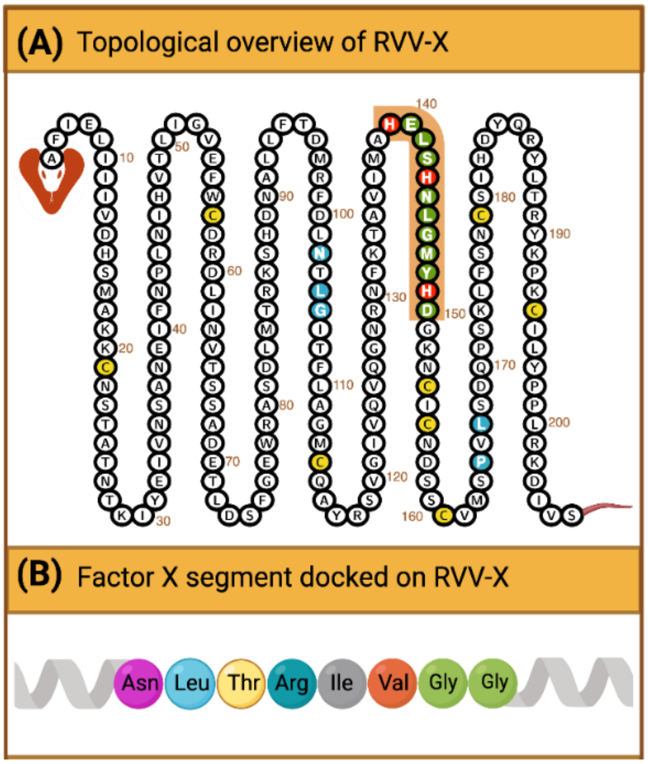

Figure S4: Schematic representation of: (A) Topological overview of RVV-X highlighting the catalytic motif (orange shadow), the cysteine residues (yellow spheres) and the binding residues (blue spheres), (B) The FX segment docked on the RVV-X. Created with BioRender.com.

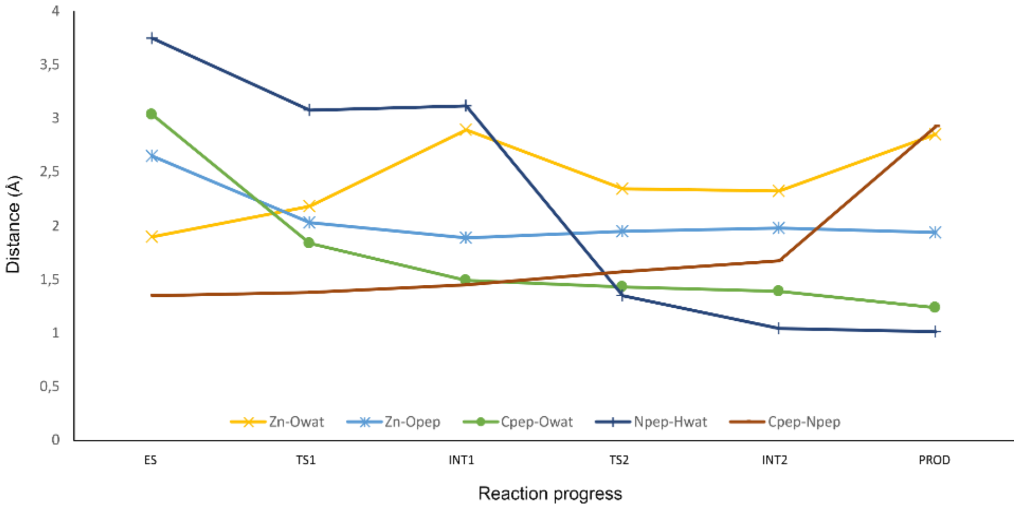

Figure S5: Key distances of the different stationary points along with the reaction progress.

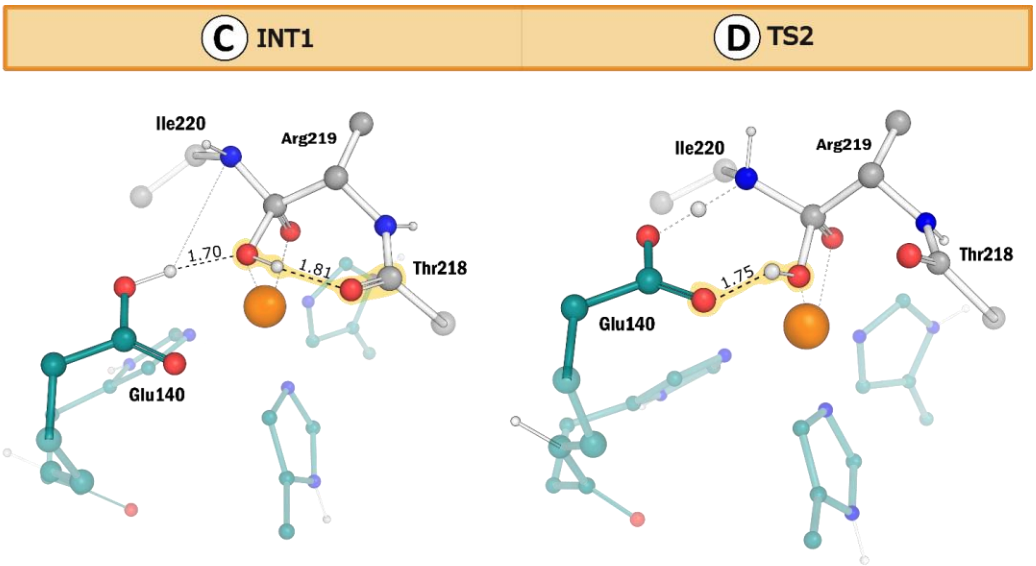

Figure S6: Representation of the hydrogen bond network changes as it progresses from (C) INT1 to (D) TS2, in which, the hydrogen bond between OHpep-OThr is replaced by a new H-bond between OHpep-Oε1Glu (yellow shadow).

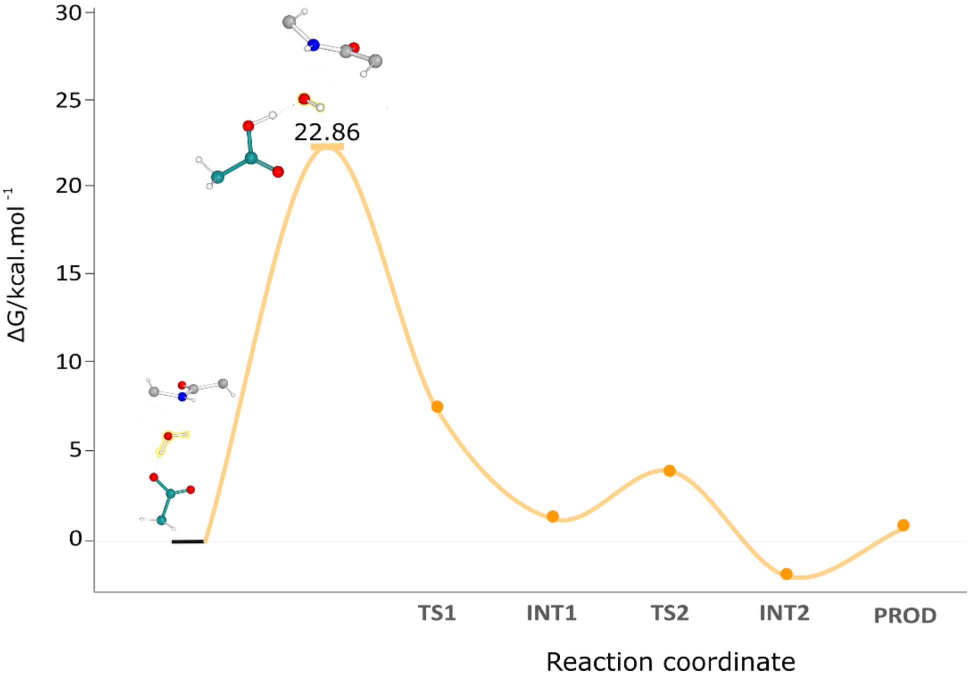

Figure S7: The ONIOM energy profile of the QM layer (B3LYP/6-31G(d)) without the cofactor showing the deprotonation of the nucleophilic water as the rate-limiting step with an ONIOM energy of 22.86 kcal·mol^{-1}. The critical residues for the first two reaction steps are shown in CPK representation.

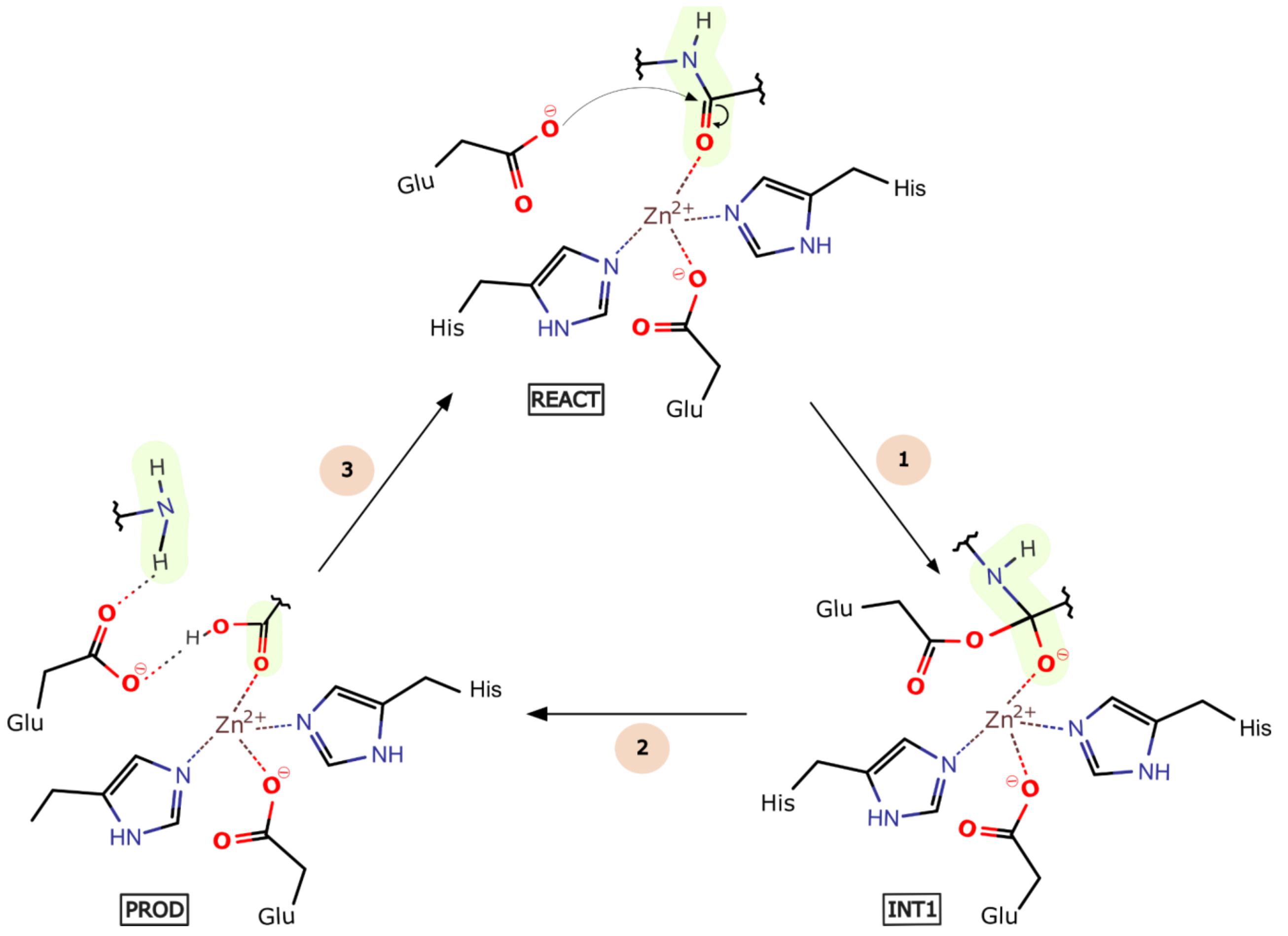

Scheme S1: The "anhydride pathway": (1) The carbonyl oxygen of the peptide is polarized by the direct binding of the Zn^{2+}, facilitating the nucleophilic attack by the unprotonated carboxylate side chain of Glu on the scissile carbonyl carbon, leading to the formation of the acyl-enzyme intermediate; A water molecule enters the active site and attacks the acyl-enzyme intermediate and protonates the amine product, resulting in the (2) product complex release; (3) Active site regeneration [1].

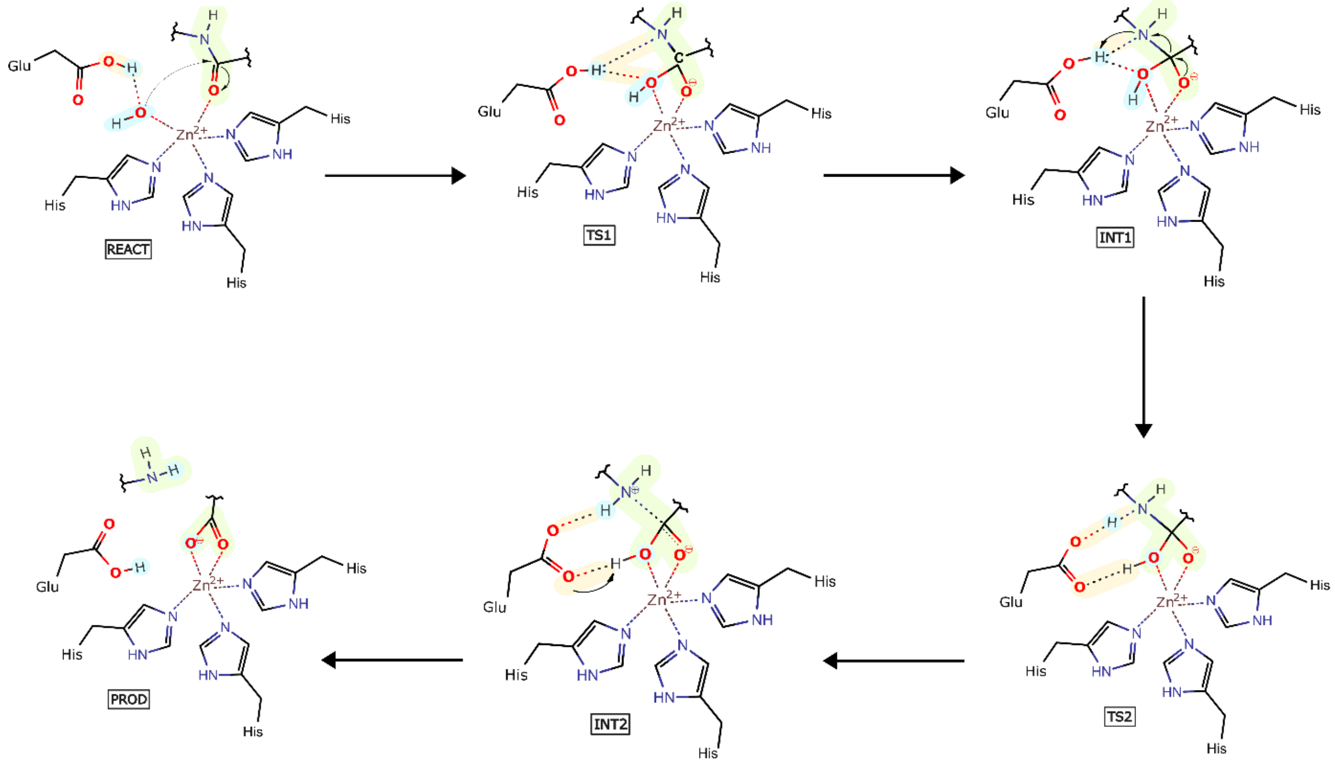

Scheme S2: Schematic representation of the final mechanism obtained for RVV-X consistent with the proposed "water-promoted pathway" mechanism.

Table S2: Electronic energies ($E_{ONIOM,\ kcal/mol}$) of each stationary state at the B3LYP, M06, and M06-L functionals with the 6-311++G(3df,2dp) basis set.

	B3LYP	M06	M06-L
**REACT**	0,0	0,0	0,0
**TS1**	16,3	13,9	14,0
**INT1**	12,5	9,2	13,2
**TS2**	21,5	17,8	21,1
**INT2**	16,5	13,8	16,0
**PROD**	-7,0	-6,3	-6,8

Computational methods

Model Construction

Mutations required to model RVV-X structure from *D. russelii*: Ile6Ala, Val11Ile, Asn74Asp, Glu122Gly, Glu126Val, Phe197Leu, Asn198Tyr, Asn229Asp, Asp271Asn, Lys296Gln, Asn326Thr.

Parametrization of the metalloproteinase's metal center

The bonded model was adopted to represent the interactions between the metal ion and the neighboring residues. Protonation states were assigned based on the pKa values predicted by the H++ web server (http://biophysics.cs.vt.edu/H++) [1,2]. However, H++ does not consider either the metal ion or the catalytic water while adding hydrogen atoms, so the protonation state of His139 was manually fixed. Besides the water molecule, the zinc ion is coordinated by three histidines, so only one nitrogen atom should be protonated, and the side chain should be neutral. For that reason, the extra "Hε2" atom was deleted, and the residue was renamed "HID" instead of "HIE". It is noteworthy mentioning that according to the general mechanism of action, the side chain of the Glu140 must be deprotonated to be capable of polarizing the catalytic water molecule and consequently receive a proton from it. The resulting PDB file was renumbered using pdb4amber.

In the next step, an MCPB.py script generated three models using the ff14SB force field, a small model used for bond and angle parameters calculation, a standard model containing the atom type information, and a large model for charge (RESP) calculation. The small model contained the zinc metal with the coordinating histidine's side chains, in which hydrogen atoms were added to the truncated bonds, whereas the bigger model possessed the smaller model with entire residues, and NME and ACE capped the truncated bonds.

Then, the B3LYP/6-31G* level of theory [3,4] was employed to perform geometry optimization and force constant calculation on the smaller model. Finally, Merz-Kollman atomic charges were calculated for the larger model with the Restricted Electrostatic Potential (RESP) method and assigned to the corresponding atoms. These calculations were carried out with the Gaussian 09 software [5].

Finally, the Seminario method, which uses the Cartesian Hessian matrix, derived the metal site force field parameters for AMBER, including the connection length, connection angle, dihedral angle, and RESP charges, and then the entire forming metal bond was integrated. MCPB.py then returned the .frcmod file necessary to generate the topology and parameters file during tLeap modeling.

Protein-peptide docking

The protein-peptide docking was done with the HPepDock online server. The software generated ten poses ranked accordingly to the lowest binding energy. After considering the interatomic distances and orientations needed for the reaction mechanism, two poses (poses **B** and **C**, with opposite orientations of the peptide, shown below) stood out as the most plausible. The peptide coordinated well with the Zn^{2+} in pose **C**; the scissile Arg219-Ile220 peptide bond was correctly oriented concerning the catalytic residues. However, in structure **B,** the orientation of the scissile Arg219-Ile220 peptide bond was nevertheless less favorable for the reaction to

occur, being more away from the catalytic glutamate. To further relax the docked structures and include conformational sampling, 5 replicas of both poses were simulated by MD during 170 ns (described in the following section). Several parameters were computed, such as the root-mean-square deviation (RMSD), the root-mean-square-fluctuation (RMSF), and critical protein-peptide interatomic distances. These MD trajectory analyses were performed using the CPPTRAJ module of the AmberTools18 package.

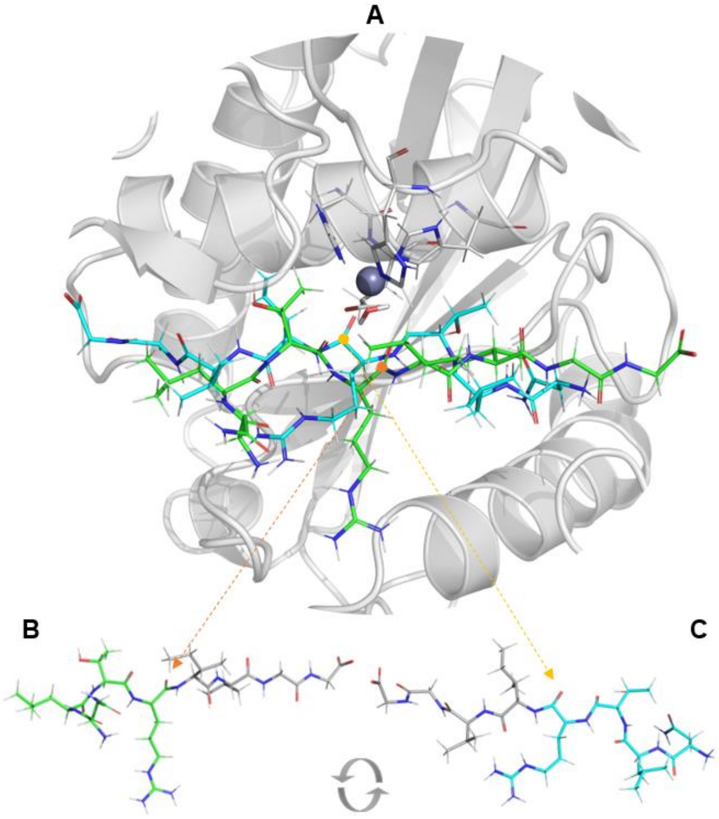

(A) Superimposition of the predicted poses for conformation **B** (green sticks) and conformation **C** (cyan stick) in the binding pocket of RVV-X protein (gray cartoon). The substrate carbonyl groups that coordinate the zinc ion are marked with a circle and arrow (orange and yellow circle and arrow, for conformation **B** and **C**, respectively). (B) Conformation **B** has an orientation that is reversed concerning ilomastat. Green and cyan sticks correspond to the activation peptide residues, while gray sticks correspond to the amino residues of the FXa domain; (C) Conformation **C** has an orientation in line with the one from the ilomastat.

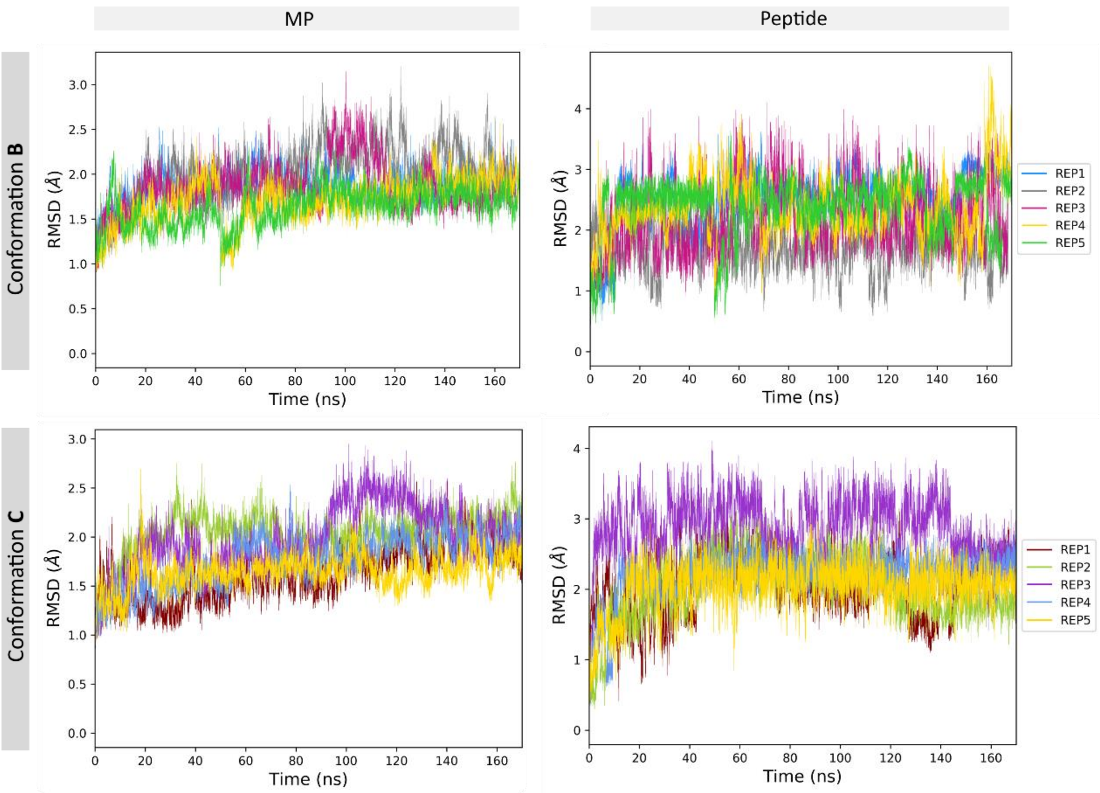

Backbone RMSD profiles as a function of time for the metalloproteinase (left) and for the peptide (right) of both conformation **B** and conformation **C** in each replica. Higher RMSD value implies low stability of the protein structure.

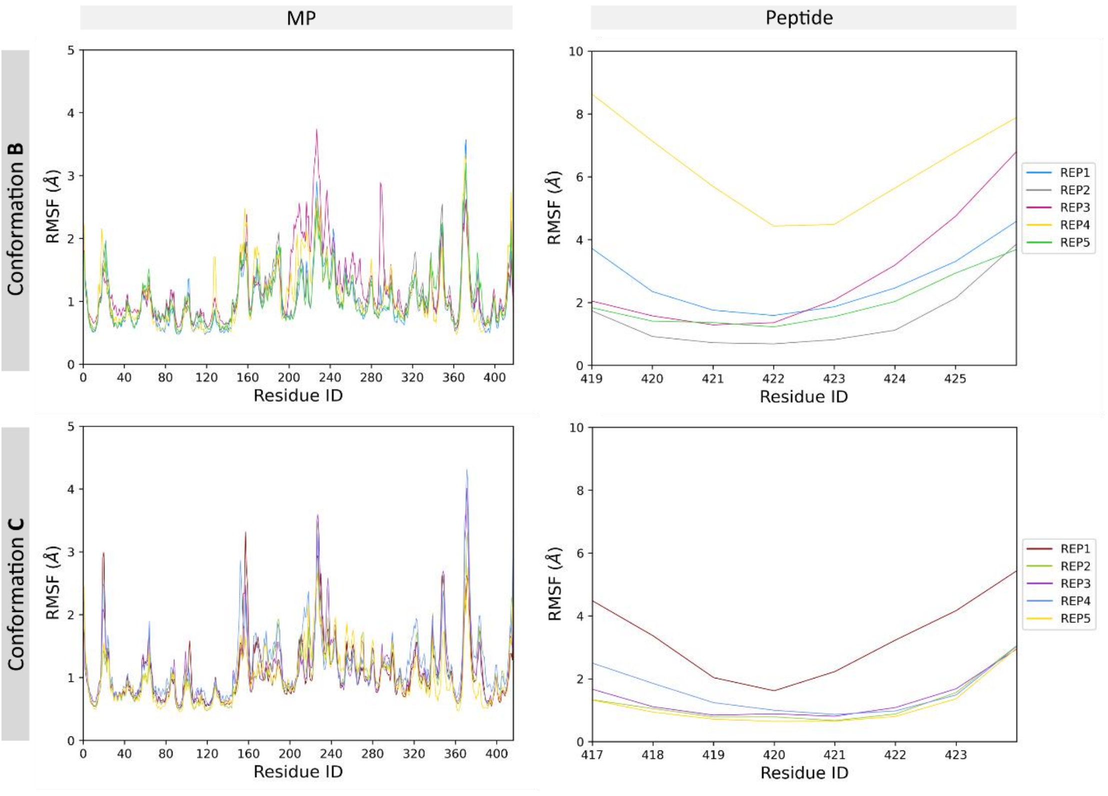

RMSF profiles of the metalloproteinase (left) and the peptide (right) for both conformation **B** and **C**. Peaks are representatives of areas of high residual flexibility. 5 replicas are represented.

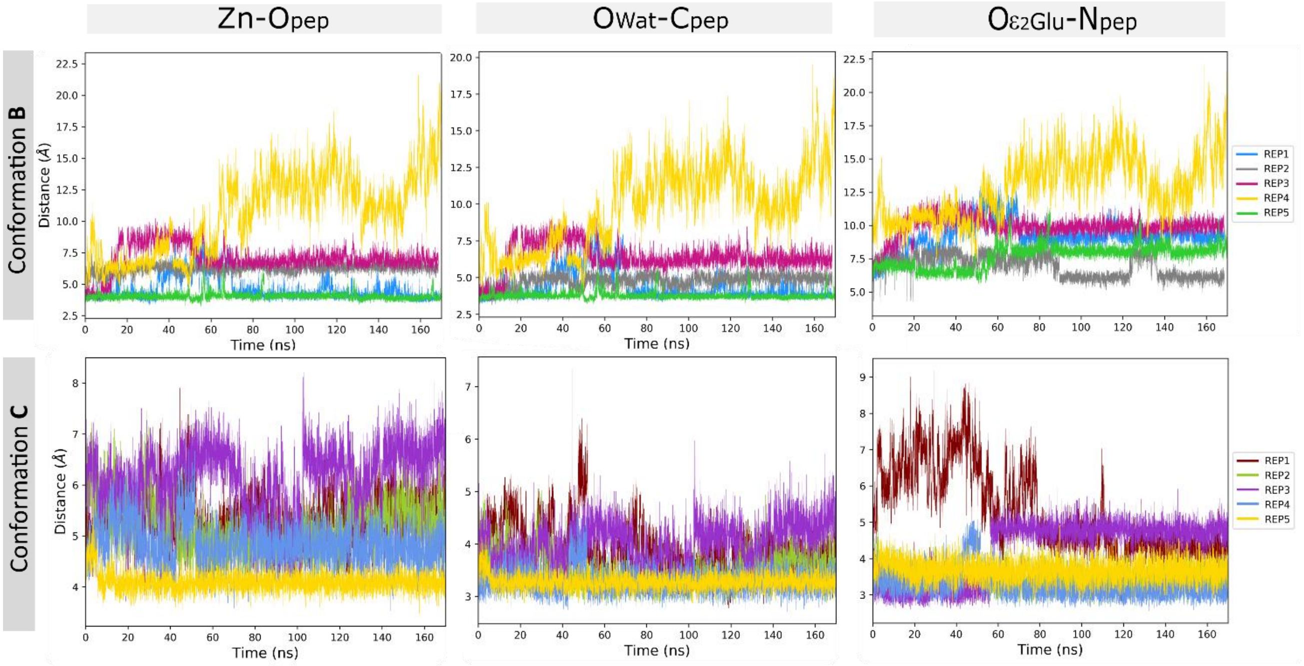

Distance distribution between the Zn ion and the carbonyl oxygen (Zn-O_{pep}), catalytic water's oxygen and the carbonyl carbon of the scissile peptide bond (O_{wat}-C_{pep}) and Gu140-amine nitrogen from the scissile bond ($O_{\varepsilon 2Glu}$-N_{pep}) for both conformation **B** and **C**. 5 replicas are represented.

The obtained results from MD confirmed that conformation **C** is far more suitable to the study of the reaction mechanism, as the substrate position was more stable and presented distances more suitable for catalysis. Finally, we compared pose **C** with the pose of Ilomastat, co-crystallized with the *D. siamensis* RVV-X. The substrate (blue sticks) and Ilomastat (red sticks) share five hydrogen bonds with the surrounding residues of the "bulge-edge segment" (Gly106, Leu105, Asn103) and the "S1'-wall forming segment" (Pro165 and Leu 167), as shown in the following figure. Furthermore, the isoleucine side chain inserts into the hydrophobic S1'-specificity pocket, almost perfectly superimposed on the Ilomastat chain (shaded in pink), while the arginine carbonyl group overlaps the Ilomastat carbonyl of the hydroxamate region (shaded in green) pointing to the Zinc ion.

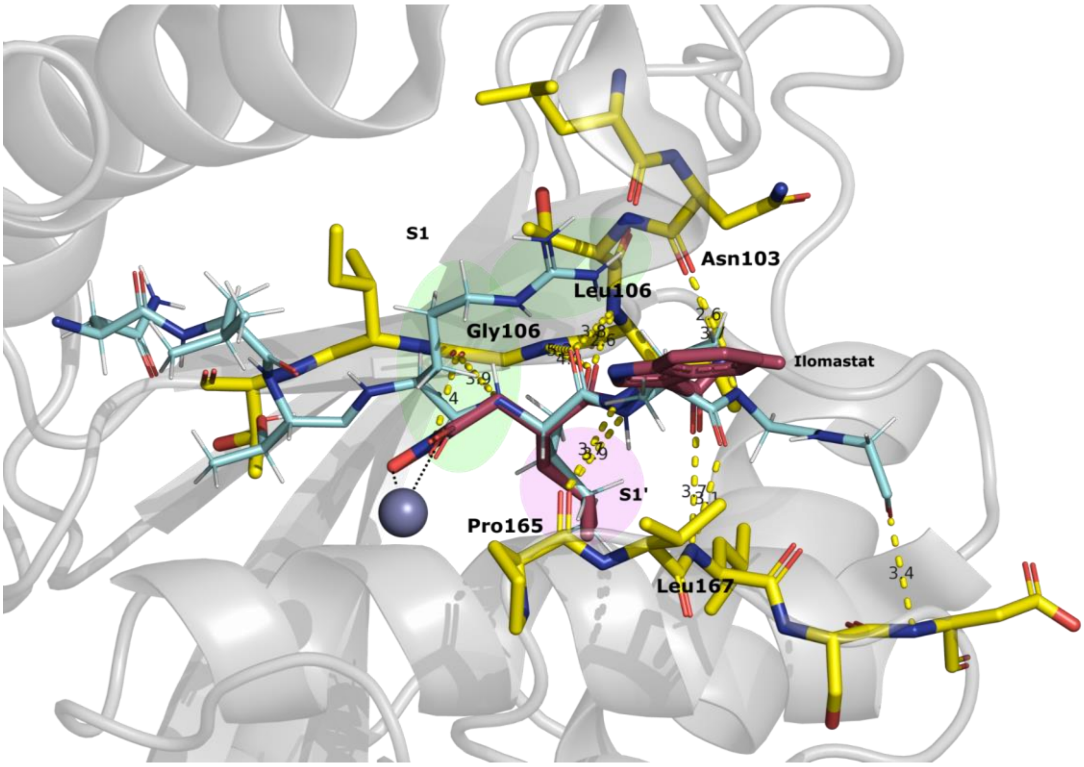

Superimposition of the chosen conformation of the peptide (blue sticks) with the Ilomastat inhibitor (red sticks) and its interactions with the surrounding residues of the protein (yellow sticks). The Zn^{2+} cofactor is represented as a slate blue sphere.

Energy minimization process

Firstly, we minimized all water molecules and then all the hydrogen atoms. Subsequently, all atoms from the side chains were minimized, and finally, an unconstrained minimization of all systems was conducted.

Molecular dynamics simulations

The molecular dynamics simulations started with all the atoms being subjected to a slow heating procedure (equilibration) in which the system's temperature gradually increased from 0 K to 300 K, using the Langevin thermostat. The heating was performed over 5 ns at constant volume, followed by 165 ns of production at constant pressure, 1 bar, recording the trajectories every 10 000 steps. The thermostat frequency of collisions was established at 1 ps^{-1}; the integration step was 1 fs. Finally, the Particle Mesh Ewald method was used with a cut-off of 10.0 Å for the intermolecular interactions. The production run occurred at constant temperature and pressure, using the Berendsen barostat considering the isothermal-isobaric ensemble (NPT), allowing a more representative system density and, therefore, a more coherent simulation of the biological phenomena under study.

On the other hand, the same molecular dynamics process was conducted during 100 ns to retrieve starting structures for the multi-PES study. The trajectory was clustered using the four reactive distances as criteria. Specifically, the Zn^{2+}-HO^-, Zn^{2+}-scissile carbonyl oxygen, HO^--scissile carbonyl carbon, and Gu140-amine nitrogen from the scissile bond. Cutoffs were used for these four distances. The cutoff values were 2.0 Å, 3.0

Å, 2.9 Å, and 4.0 Å respectively, and were applied after a pre-selection and geometry optimization. These conformations are the most reactive, and the ones used in the calculations were taken randomly within this subset.

REFERENCES

(1) Anandakrishnan, R.; Aguilar, B.; Onufriev, A. V. H++ 3.0: automating p K prediction and the preparation of biomolecular structures for atomistic molecular modeling and simulations. *Nucleic Acids Res.* **2012**, *40* (W1), W537-W541. DOI: 10.1093/nar/gks375.

(2) Gordon, J. C.; Myers, J. B.; Folta, T.; Shoja, V.; Heath, L. S.; Onufriev, A. H++: a server for estimating p K as and adding missing hydrogens to macromolecules. *Nucleic Acids Res.* **2005**, *33* (suppl_2), W368-W371. DOI: 10.1093/nar/gki464.

(3) Beck, A. D. Density-functional thermochemistry. III. The role of exact exchange. *J. Chem. Phys* **1993**, *98* (7), 5648-5646. DOI: 10.1063/1.464913.

(4) Dill, J. D.; Pople, J. A. Self- consistent molecular orbital methods. XV. Extended Gaussian- type basis sets for lithium, beryllium, and boron. *J. Chem. Phys.* **1975**, *62* (7), 2921-2923. DOI: 10.1063/1.430801.

(5) Frisch, M. J. T., G. W.; Schlegel, H. B.; Scuseria, G. E.; Robb, M. A.; Cheeseman, J. R.; Scalmani, G.; Barone, V.; Petersson, G. A.; Nakatsuji, H.; Li, X.; Caricato, M.; Marenich, A.; Bloino, J.; Janesko, B. G.; Gomperts, R.; Mennucci, B.; Hratchian, H. P.; Ortiz, J. V.; Izmaylov, A. F.; Sonnenberg, J. L.; Williams-Young, D.; Ding, F.; Lipparini, F.; Egidi, F.; Goings, J.; Peng, B.; Petrone, A.; Henderson, T.; Ranasinghe, D.; Zakrzewski, V. G.; Gao, J.; Rega, N.; Zheng, G.; Liang, W.; Hada, M.; Ehara, M.; Toyota, K.; Fukuda, R.; Hasegawa, J.; Ishida, M.; Nakajima, T.; Honda, Y.; Kitao, O.; Nakai, H.; Vreven, T.; Throssell, K.; Montgomery, J. A., Jr.; Peralta, J. E.; Ogliaro, F.; Bearpark, M.; Heyd, J. J.; Brothers, E.; Kudin, K. N.; Staroverov, V. N.; Keith, T.; Kobayashi, R.; Normand, J.; Raghavachari, K.; Rendell, A.; Burant, J. C.; Iyengar, S. S.; Tomasi, J.; Cossi, M.; Millam, J. M.; Klene, M.; Adamo, C.; Cammi, R.; Ochterski, J. W.; Martin, R. L.; Morokuma, K.; Farkas, O.; Foresman, J. B.; Fox, D. J. Gaussian 09. Gaussian Inc., Wallingford CT: 2009.
